# Miscibility and Cocrystallization in Ethylene–Vinyl Alcohol Copolymer Blends

**DOI:** 10.1021/acsapm.6c01860

**Published:** 2026-06-09

**Authors:** Asmita Ghosh, Richard A. Register

**Affiliations:** Department of Chemical and Biological Engineering, 6740 Princeton University, Princeton, New Jersey 08544-5263, United States

**Keywords:** Polymer Blend, Miscibility, Cocrystallization, EVOH, Thin Film, Mixing Thermodynamics

## Abstract

The compatibility among different ethylene–vinyl alcohol (EVOH) copolymer grades plays a crucial role in determining how easily they can be recycled when present together in a mixed waste stream. In this work, melt miscibility and cocrystallization in 50/50 wt/wt binary blends of EVOH copolymers with differing ethylene content were studied. Blends with up to a 21 mol % difference in composition were analyzed. Thin films of miscible blends appeared homogeneous in the melt as observed under an optical microscope, whereas AFM height images showed that immiscible blends underwent surface roughening due to phase separation. For copolymers with modest composition differences, the blend behaved as a single component, showing single, narrow melting and crystallization peaks in DSC thermograms, indicating cocrystallization at the level of crystal stems. Interestingly, even phase-separated blends demonstrated partial cocrystallization due to partial miscibility of the components in the molten phase-separated domains. The extent of cocrystallization is governed by the difference in freezing points of the components; rapid cooling increased cocrystallization in the blends. The maximum compositional difference allowed for miscibility is not constant but depends on the blend’s average comonomer content, in agreement with the “miscibility window” calculated from literature pressure–volume–temperature behavior for the individual EVOH copolymers and regular mixing theory. This study helps illuminate the effect of copolymer composition on miscibility, as well as cocrystallization behavior in blends of random copolymers with cocrystallizable units.

## Graphical abstract

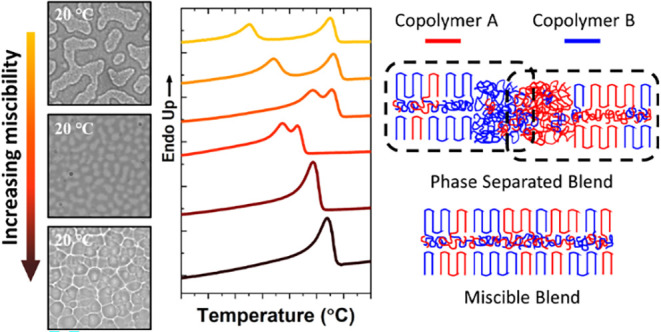

## 1 Introduction

Ethylene–vinyl alcohol (EVOH) copolymers provide excellent barrier against oxygen permeation and are widely used in multilayered packaging materials. The incompatibility of different layers like EVOH and polyethylene (PE) renders the recycling of such complex structures a major challenge.^1–3^ EVOH copolymers are reported to be immiscible with poly­(vinyl alcohol) (PVOH) as well.^4–6^ Though immiscible with both PE and PVOH, EVOH copolymers of differing ethylene content should be miscible with each other if the composition difference is sufficiently small. Commercially available EVOH copolymer grades span a range of ethylene contents. As sustainability considerations increasingly shape materials design, polymer recyclability has emerged as a central scientific and technological challenge. Mechanical recycling of incompatible polymers typically produces materials with inferior mechanical performance due to sharp interfaces between phase-separated domains.^7^ In contrast, compatible polymer systems, even when phase-separated, exhibit sufficient interchain entanglement across the interface to retain good mechanical properties.^8,9^ Although considerable effort has been devoted to studying compatibility in diverse polymer blends, to the best of the authors’ knowledge, no systematic studies have addressed the compatibility among EVOH copolymers of differing ethylene content. This is particularly consequential in light of the projected growth of the global EVOH market, which is expected to expand at a compound annual growth rate of 6.5% between 2026 and 2035.^10^ As a result, various EVOH grades are increasingly likely to enter mixed waste streams, making an understanding of their mutual compatibility critical for effective recycling. Mechanical compatibility between two polymers is often enhanced by their ability to cocrystallize, a phenomenon that occurs only under stringent conditions such as similarity in repeat unit chemistry, comparable lattice parameters, and partial melt miscibility^11^—criteria that EVOH copolymers are well-positioned to satisfy. Apart from mechanical recycling, recent developments in the recycling of multilayer structures include the solvent-targeted recovery and precipitation (STRAP) process, in which selective solvents are employed to dissolve and separate individual layers.^12^ In such processes, multiple EVOH copolymer grades are likely to coexist within a single stream, as they all exhibit solubility in common solvents such as dimethyl sulfoxide (DMSO).^12^ Mechanical compatibility is also necessary for packaging materials formed from EVOH copolymers with a bimodal composition distribution, to achieve good oxygen barrier properties at elevated relative humidities.^13,14^ A recent study demonstrated that blends of two stereoisomeric analogs of EVOH undergo phase separation during crystallization, as the diastereomers fail to cocrystallize due to disparities in their crystal structures and crystallization kinetics, a behavior that can be used to tailor barrier and mechanical properties.^15^ Therefore, insight into the phase behavior and cocrystallization ability of blends comprising EVOH copolymers of different compositions is of critical importance.

The present study investigates the phase behavior of binary blends comprising EVOH copolymers with different ethylene contents. Within the composition range studied, the copolymers exhibit closely spaced glass transition temperatures (*T*_g_), making it difficult to determine miscibility from the composition dependence of *T*_g_. Moreover, crystallization in melt-miscible semicrystalline polymer blends may lead to liquid–solid phase separation, giving rise to unusual glass transition behavior.^16,17^ This renders conventional assessments of miscibility based on *T*_g_ inconclusive. In addition, the shared comonomer chemistry among the EVOH grades limits the applicability of several commonly employed miscibility tests. For instance, EVOH copolymers have similar refractive index, so even immiscible blends will not scatter light intensely. However, thin films of multicomponent fluids often undergo surface roughening due to lateral phase separation, and this roughening mirrors the phase separation in the bulk material,^18–20^ a phenomenon that has been exploited in this study to investigate blend phase behavior.

The primary objective of this work is to determine miscibility and cocrystallization in blends of EVOH copolymers spanning the commercial composition range as functions of comonomer content. Immiscible EVOH blends were found to undergo surface roughening during phase separation, resulting in a distinct pattern of height variations in the thin (order ∼ 1 μm) polymer films. For blends with large composition differences, the height variations were of the order of the film thickness. EVOH copolymers were found to be miscible within a finite composition window, with the critical composition difference for miscibility dependent on the average ethylene content in the blend. At room temperature, across the entire composition range, EVOH copolymers are semicrystalline, with both E and VOH units capable of incorporating into the crystal lattice.^21^ The extent of cocrystallization between blend components was systematically evaluated using thermal analysis. A miscibility–cocrystallization map as a function of ethylene content was constructed, and the experimental miscibility behavior was evaluated against predictions from regular mixing theory.

## 2 Experimental Section

### 2.1 Materials

The EVOH copolymers used in this study were obtained from different sources along with their compositions; their melt indices (MI, g/10 min) are all reported under 2160 g load, and at 190 °C unless otherwise specified. EVOH copolymers of 38 and 27 mol % ethylene content were purchased from Scientific Polymer Products Inc., with MI values measured to be 2.33 and 4.67 (the latter at 210 °C), respectively. Experimental EVOH copolymers of 44 and 32 mol % ethylene content were obtained from Kuraray, and their MI values were measured to be 5.44 and 1.31, respectively. Commercial EVOH copolymers with 48 mol % ethylene (EVAL G176B, nominal MI = 6.5) and 35 mol % ethylene (EVAL C109B, nominal MI = 8.5) were also provided by Kuraray. The EVOH copolymers are designated here as EVOHXX, where XX is the mole percent ethylene. DMSO was used as received.

### 2.2 Melt Blending Copolymers

50/50 wt/wt binary blends of EVOH copolymers were melt-blended in a Thermo Scientific HAAKE MiniLab II microcompounder with counter-rotating conical twin screws and an integrated backflow channel. The copolymers were dried at 65 °C for 24 h in a vacuum oven prior to processing. The blending temperature was kept ∼15 °C above the peak melting temperature of the higher-melting component. The screw speed was kept at 45 rpm. The backflow channel enabled recirculation of the polymer melt through the compounder, and the copolymers were blended for 30 min prior to extrusion. The extrudates were melt-pressed between poly­(ethylene terephthalate) sheets into films 0.1–0.3 mm thick, at least 10 °C above their melting temperatures. The films were either quenched from the melt by submerging the sandwich in tap water (cooling rate ∼ 1000 °C/min) or slowly cooled (cooling rate ∼ 1 °C/min) by leaving the film to crystallize overnight in the hot press as the platens cooled. The EVOH copolymer blends are designated by EVOHXX-YY, where XX and YY are the mole percent ethylene of each component. To take into account the effect of melt blending and extrusion on the EVOH copolymers, the individual components were also processed under similar conditions and blend properties were compared with those of the extruded copolymers. The processing temperatures for the individual components and blends are given in Tables S1 and S2 respectively.

### 2.3 Optical Microscopy

For optical microscopy studies, films of average thickness in the range of 0.8–3 μm were made by drop casting from an EVOH blend solution in DMSO, at a total copolymer concentration of 10 mg/mL, onto borosilicate micro coverslips. Prior to drop casting, the surface of the coverslips was cleaned with acetone. DMSO was removed under vacuum at 80 °C for a minimum of 48 h. The surface of the film was kept uncovered (free), and the evolution of the domain morphology of the blends was observed under a Zeiss Axio Scope.A1 optical microscope (OM) in transmission mode. The microscope was equipped with a Linkam stage (FTIRSP 600 with T96-S controller) used to control the temperature of the films during melting and crystallization. The films were heated to 220 °C at 10 °C/min and held for at least 15 min to observe domain coarsening. After that, the films were cooled to 20 °C at 100 °C/min. Crystal superstructures (e.g., spherulites) were observed under 90° crossed polarizers. The captured OM images were edited to enhance the sharpness and contrast and the saturation was set to zero for uniform presentation. Correlation length was determined from an 80 μm × 80 μm area cropped from the bright-field OM image and scaled to 256 × 256 pixel size, using a Python code to calculate the one-dimensional (1D) autocorrelation function.

### 2.4 Atomic Force Microscopy

The film surface topography was characterized by atomic force microscopy (AFM) using a Bruker NanoMan in tapping mode. Antimony (n) doped Si AFM probes (force constant = 40 N/m, resonance frequency = 300 kHz) purchased from Bruker were used for measurements. Height images of the specimens prepared for OM (melted and cooled) were acquired to observe the topography of the film surface after evolution of domain morphology. All images were taken at 256 × 256 pixel resolution with scan size ranging from 20 μm × 20 to 80 μm × 80 μm. The 1D autocorrelation function was calculated on the as-obtained height images.

### 2.5 Differential Scanning Calorimetry

A PerkinElmer DSC 7 was used to measure the thermal properties of the molded films. Aluminum pans were used for the sample and reference. The instrument was calibrated for temperature using indium and tin, and for enthalpy using indium. Two empty pans were scanned under the same protocol as the sample to determine the calorimeter baseline, which was then subtracted from the sample data. The heating and cooling rates were set to 10 °C min^–1^ unless stated otherwise. Samples were heated from a temperature at least 20 °C below their *T*_g_ to at least 15 °C above the peak melting temperatures, *T*_m_. For isothermal crystallization experiments, samples were held at the given temperature for 60 min.

### 2.6 Wide Angle X-ray Diffraction

Wide-angle X-ray diffraction patterns of the molded films were recorded at room temperature using a Bruker D8 Discover X-ray Diffractometer in the Bragg–Brentano θ–2θ geometry. Ni-filtered Cu–Kα radiation (λ = 0.15418 nm) was used. The diffraction scans were collected from 5° to 40° in 2θ at intervals of 0.04° with a scan rate of 1.5° min^–1^.

## 3 Results and Discussion

A total of 15 binary blends were prepared by melt-blending, listed in Table S2. It has been reported that EVOH copolymers undergo degradation at temperatures close to their melting point.^22–24^ The process of melt-blending can therefore affect the thermal and mechanical properties of these copolymers. To compare the thermal properties of the blends with the individual components, the individual copolymers were processed under similar conditions. The effect of extrusion on the thermal properties is shown in Figures S1–S3. The individual components processed similarly showed a 1–4 °C decrease in the melting and freezing temperatures due to the compounding process. Torque values did not change significantly during the mixing time (30 min, 45 rpm screw speed) implying no significant chain scission or cross-linking. The EVOH blends were classified as either miscible or phase-separated as described below.

### 3.1 Miscible Blends

Miscibility of the EVOH copolymer blends was examined by bright-field optical microscopy on drop-cast thin (∼1 μm) films, as shown in Figures 1(a) and S4–S12. A blend was considered miscible if it exhibited a homogeneous texture in the melt similar to that of the constituent copolymers. Figure S13 shows how an individual EVOH copolymer (EVOH35) appeared in the melt and upon crystallization. Thin films of individual EVOH copolymers did not undergo dewetting or film rupture upon melting. Figure S16 also shows the AFM height images of EVOH27 and EVOH35, where there is no evidence of any film rupture.

**Figure 1:**
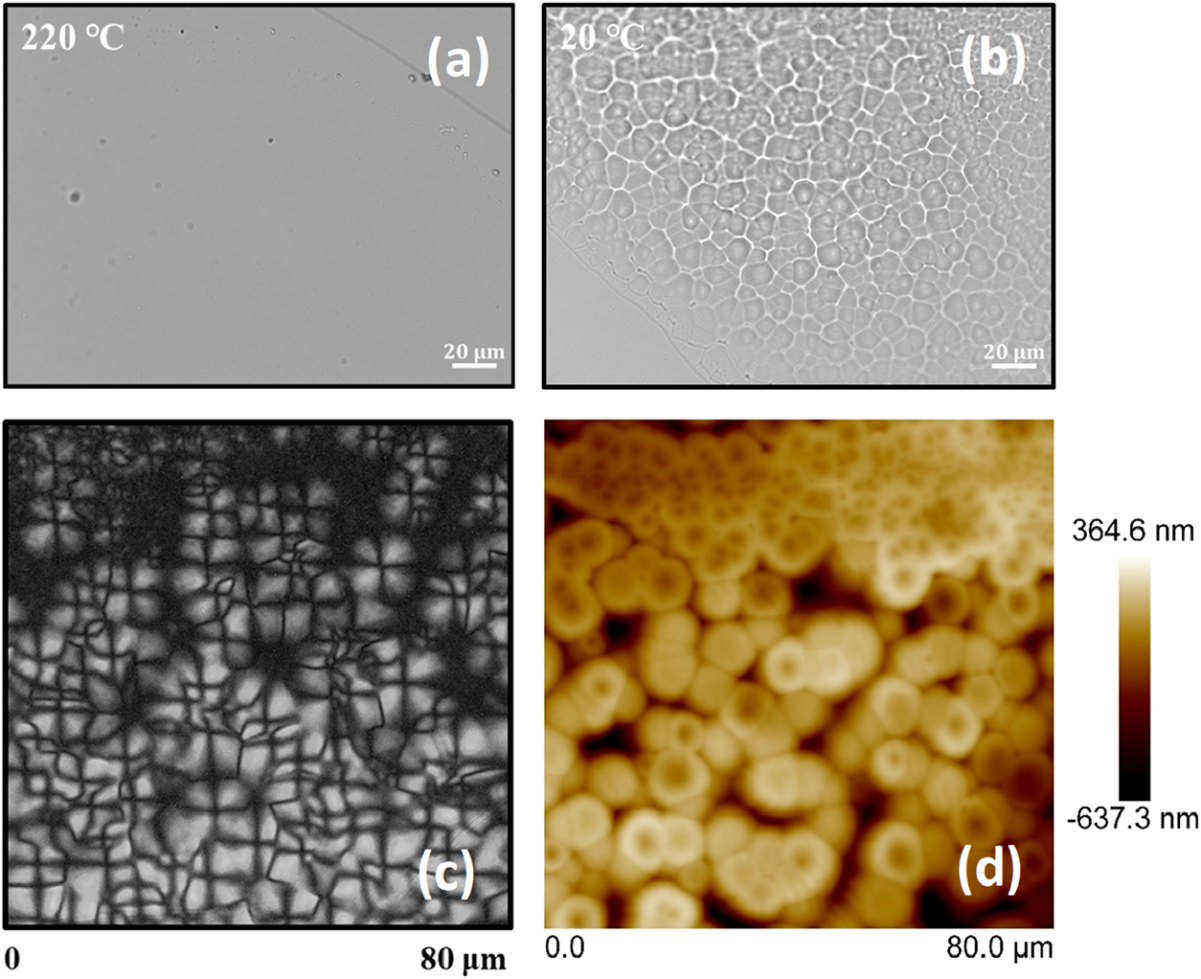
EVOH38–32 blend showing (a) homogeneous melt state (film appears uniform) under bright-field OM; (b) spherulites formed upon cooling to 20 °C; (c) POM image (80 μm × 80 μm) of spherulites at 20 °C; (d) Room-temperature AFM height image (80 μm × 80 μm) showing structures of similar length scale. The heating and cooling rates were 10 °C/min and 100 °C/min, respectively.

By virtue of the ability of both E and VOH comonomer units to incorporate into the crystal lattice, and the similarity in lattice parameters among the copolymers,^21^ EVOH copolymers of differing composition are likely to cocrystallize with one another. EVOH38–32, a miscible blend, has a composition difference (Δ*f*_e_) of 6 mol %. In Figure 1(a), EVOH38–32 appears homogeneous in the melt by bright-field optical microscopy. Figure 1(c) shows the formation of spherulites due to crystallization of the components upon cooling to 20 °C, as observed by polarizing optical microscopy (POM). AFM height images of the EVOH38–32 thin film (Figures 1(d) and S17) exhibit spherulitic features with characteristic length scales comparable to those observed by POM, similar to individual copolymers (Figure S16­(b)). These observations indicate that EVOH38–32 is miscible in the melt and undergoes crystallization upon cooling without any evidence of phase separation. Figure 2 shows the DSC thermograms of EVOH38–32. In Figure 2(a) and subsequent figures presenting DSC heating curves, the solid lines are the second heating traces, after cooling at 10 °C/min from the melt. The first heating traces (dashed lines) are shown for slowly cooled (SC) samples that were crystallized upon cooling at ∼1 °C/min, and for quenched (*Q*) samples that were cooled at ∼1000 °C/min, both prior to loading into the DSC. The DSC heating and cooling thermograms of EVOH38–32 all exhibit single, sharp melting and crystallization peaks, indicating blend miscibility and complete cocrystallization at the level of crystal stems over all practically relevant cooling rates. The melting and freezing temperatures of the EVOH38–32 blend, which has an average ethylene content of 35 mol %, are comparable to those of the EVOH35 copolymer processed under similar conditions (Figure S24). Furthermore, the X-ray diffraction patterns of the EVOH38–32 blend and EVOH35 copolymer also coincide (Figure S24), proving that the blend cocrystallizes completely, behaving as a single copolymer of intermediate composition. Other blends falling into this category include EVOH32–27, EVOH35–32, and EVOH38–35. The DSC thermograms of these blends are shown in Figures S21–S23.

**Figure 2:**
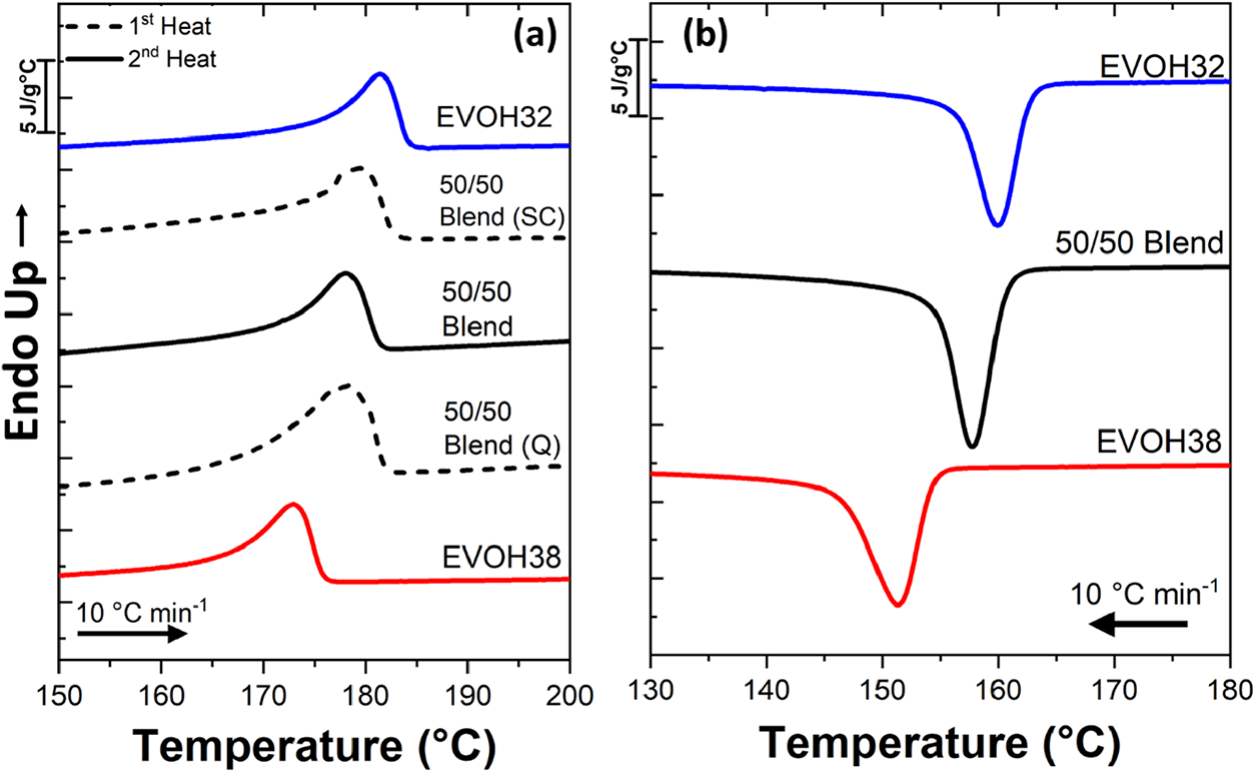
DSC thermograms of EVOH38–32 compared with individual components processed at 200 °C: (a) heating curves; (b) cooling curves.

### 3.2 Blends Showing Phase Separation

Blends with larger composition differences exhibited clear phase separation in the melt, as evidenced by optical microscopy. The OM images of the EVOH48–27 (Δ*f*_e_ = 21 mol %) thin film revealed distinct features in the molten state (Figure 3(a)) that persisted after quickly cooling to 20 °C (Figure 3(b)). As these features were absent in the miscible blends as well as in the individual components, dewetting was ruled out, and phase separation was identified as the origin of the observed morphology. The domains were observed to coarsen with time (Figures S14 and S15), consistent with phase separation. However, the coarsening process slowed markedly at longer times (after 15 min) due to cross-linking^22^ in the EVOH copolymers at 220 °C, which limited further domain growth. The EVOH27 film held at 220 °C for over 15 min no longer dissolved completely in DMSO, confirming cross-linking. As a result, large domains were not always observed, leading to a wide range of average domain sizes across the blends exhibiting phase separation (Figures S4–S6 and S8–S12). However, due to the similar chemical structure of the components, determining the composition of the domains was not possible. To further elucidate the origin of the features observed in optical microscopy for the phase-separated blends, AFM was employed.

**Figure 3:**
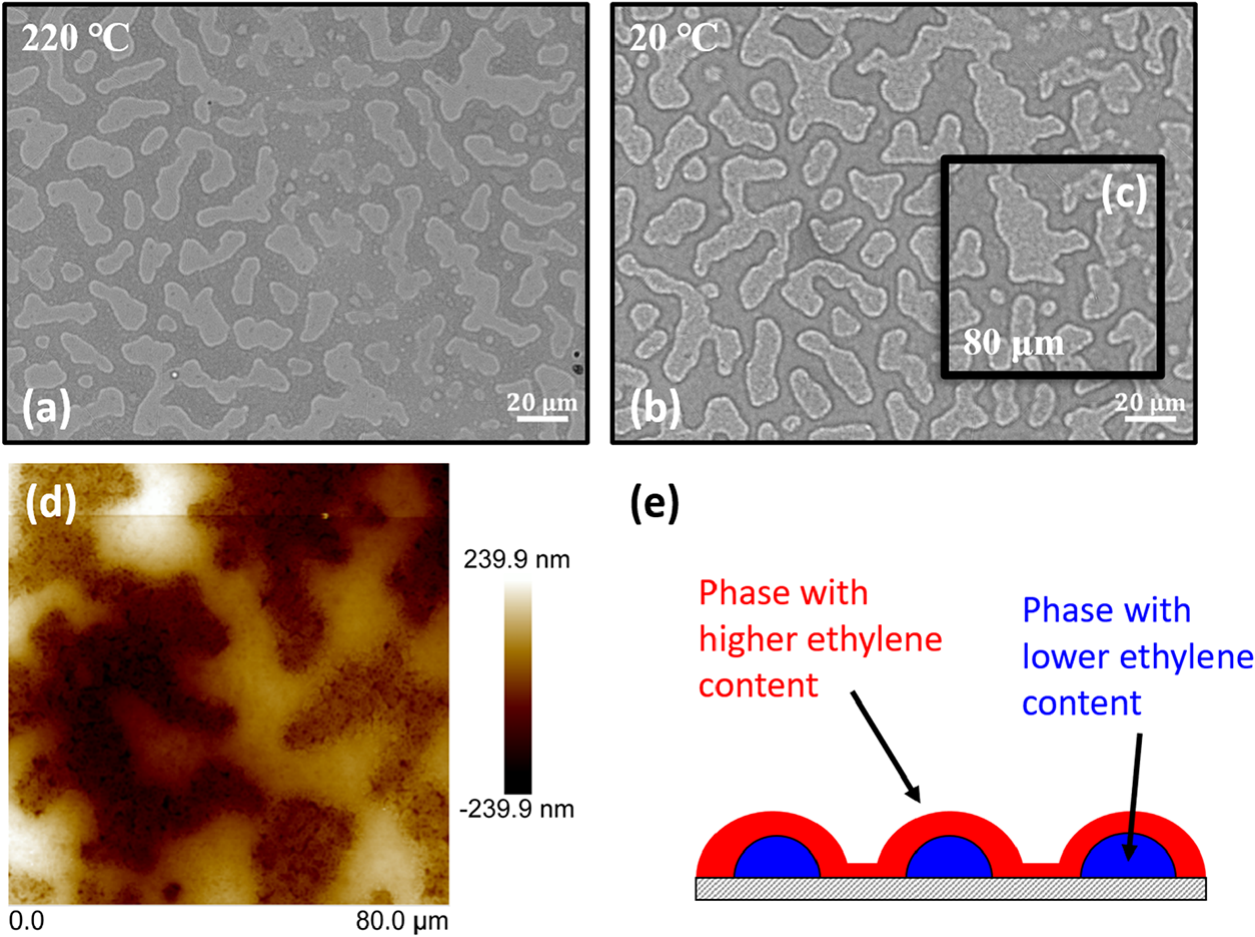
Bright-field OM images showing EVOH48–27 (a) undergoing phase separation in the melt and (b) after cooling; inset (c) shows an 80 μm × 80 μm snippet of (b). (d) AFM height image of EVOH48–27 showing topography of the film surface, due to lateral phase separation. (e) Schematic cross-section of domain structure of a phase-separated blend film showing surface roughening.

The AFM height images showed pronounced topographical variations in the phase-separated blends, with the length scale of the domains comparable to that observed by optical microscopy (Figure S19). This indicates that phase separation in the blends induces surface roughening, and that the contrast observed in optical microscopy arises primarily from optical path length variations introduced as a result of surface undulations of the phase-separated film,^20^ rather than from refractive index differences between coexisting phases within a film of uniform thickness. The AFM height image of the EVOH48–27 blend (Figure 3(d)) clearly reveals phase separation, where the domain shape and size are comparable to the features observed in the OM image (Figure 3(c)). The bright regions in the OM images correspond to low regions (valleys) in the AFM height images.

The topographic variation observed by AFM (Figure 3(d)) reflects lateral phase separation, as schematized in Figure 3(e). The surface tension of EVOH copolymers decreases with increasing ethylene content;^25^ we thus infer that the film free surface is everywhere enriched in the component with higher ethylene content, covering both the island and valley regions. The component with higher vinyl alcohol content is expected to have an affinity for the glass substrate; for example, the work of adhesion between polymer and glass increases with vinyl alcohol content in vinyl butyral-vinyl alcohol copolymers.^26^ Thus, one might expect that the equilibrium structure of a phase-separated blend of EVOH copolymers would be a smooth bilayer, with a higher-ethylene layer at the surface and a lower-ethylene layer at the glass substrate. However, the equilibrium film morphology is dictated by the collective values of the component surface tensions, the intercomponent interfacial tension, and the tension between each component and the substrate.^19,20,27–29^ A smooth bilayer is favored when the intercomponent interfacial tension is small, but for some combinations of tensions, the bilayer structure is unstable relative to lateral component segregation.^19,27^ While cross-linking of the EVOH at elevated temperatures complicates assessment of the equilibrium structure, the fact that the film is initially smooth, and spontaneously roughens upon heating (Figure S18), strongly suggests that the island/valley structure schematized in Figure 3(e) is thermodynamically favored.

An important observation was that as the incompatibility between the components decreased, the domain sizes generally decreased, as shown in Figure 4. The correlation lengths of domains observed in the melt (220 °C), obtained from the OM images of the phase-separated blends, are shown in Figure S20. As the interfacial tension between the components decreases, the driving force for domain growth weakens and the rate of domain coarsening slows, such that only smaller domain sizes are achieved by the time cross-linking arrests domain coarsening. AFM height images show the same trend (Figure S18).

**Figure 4:**
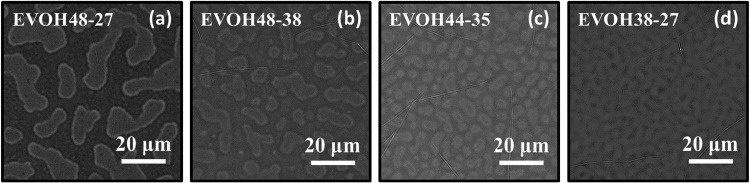
(a–d) OM images (80 μm × 80 μm) of different EVOH blends as indicated, at 220 °C, after holding for 15 min.

### 3.3 Cocrystallization in EVOH Blends

As noted earlier, miscible EVOH blends can undergo cocrystallization at the level of crystal stems upon dynamic cooling at 10 °C/min, as evidenced by the presence of a single melting/crystallization peak in the DSC thermograms. These systems were categorized as cocrystallizing blends. However, the extent of cocrystallization depends on the difference in freezing points of the components (Δ*T*_f_) and the cooling rate; under certain conditions, partial cocrystallization of the components occurs.

#### 3.3.1 Partial Cocrystallization Observed in Miscible Blends at Slower Cooling Rate

As Δ*T*_f_ becomes greater than 7 °C, even miscible blends show only partial cocrystallization upon slowly cooling from the melt. Figure 5(a) shows that the EVOH35–27 blend (Δ*T*_f_ = 11 °C) appears homogeneous in the melt, indicating melt miscibility. When cooled rapidly, at 100 °C/min, the blend crystallized to form spherulites as shown in Figures 5(b) and (c). When cooled in the DSC at 10 °C/min, only a single, narrow exotherm is observed (Figure S25), indicating complete cocrystallization. However, in Figure 5(d) the DSC heating traces show different melting behavior for different thermal treatments. Upon quenching from the melt, the blend shows complete cocrystallization, as evidenced by the single endotherm. By contrast, the first heating trace of the slowly cooled EVOH35–27 blend exhibited an additional endothermic peak at a temperature lower than that of EVOH35, appearing adjacent to the main melting endotherm, which lies between the melting temperatures of the individual components. The emergence of this lower-temperature peak was attributed to the inability of EVOH35, which has a lower freezing temperature, to fully cocrystallize with EVOH27, resulting in separate crystallization of a fraction of EVOH35 upon slow cooling. Similar results were observed in the miscible EVOH48–44 blend (Figure S26). This shows that even though miscible, due to a larger difference in freezing points, the components start crystallizing separately as the cooling rate is reduced.

**Figure 5:**
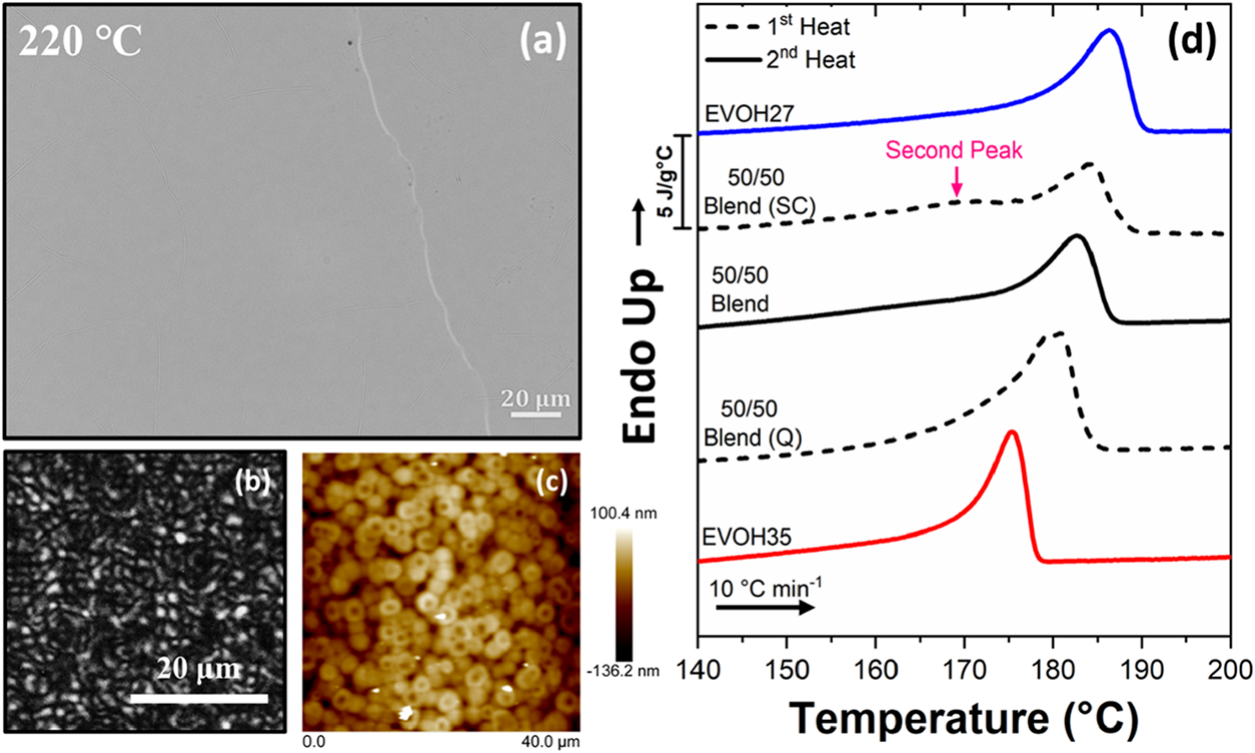
(a) EVOH35–27 appears uniform in the melt (miscible) by bright-field OM; (b) POM image (40 μm × 40 μm) showing crystallization upon cooling to 20 °C at 100 °C/min; (c) AFM height image showing spherulitic structures; (d) DSC heating traces of the blend compared with EVOH35 and EVOH27.

#### 3.3.2 Partial Cocrystallization in Phase-Separated Blends

Figure 6 shows the DSC thermograms of the phase-separated blend EVOH38–27 (Δ*T*_f_ = 14 °C). The melting and crystallization behavior of EVOH38 in the blend differs from that of EVOH38 processed under similar conditions. The elevated melting (*T*_m_) and freezing (*T*_f_) temperatures of EVOH38 in the blend suggest partial cocrystallization with EVOH27, giving rise to two distinct crystal populations: one consisting essentially of EVOH27 alone, and the other primarily composed of EVOH38 but incorporating a fraction of EVOH27. These results indicate that, even in phase-separated systems, the domains are not composed of the individual component copolymers A and B, but rather of A-rich and B-rich phases that can retain the ability to cocrystallize at the level of crystal stems, at least under sufficiently rapid crystallization.

**Figure 6:**
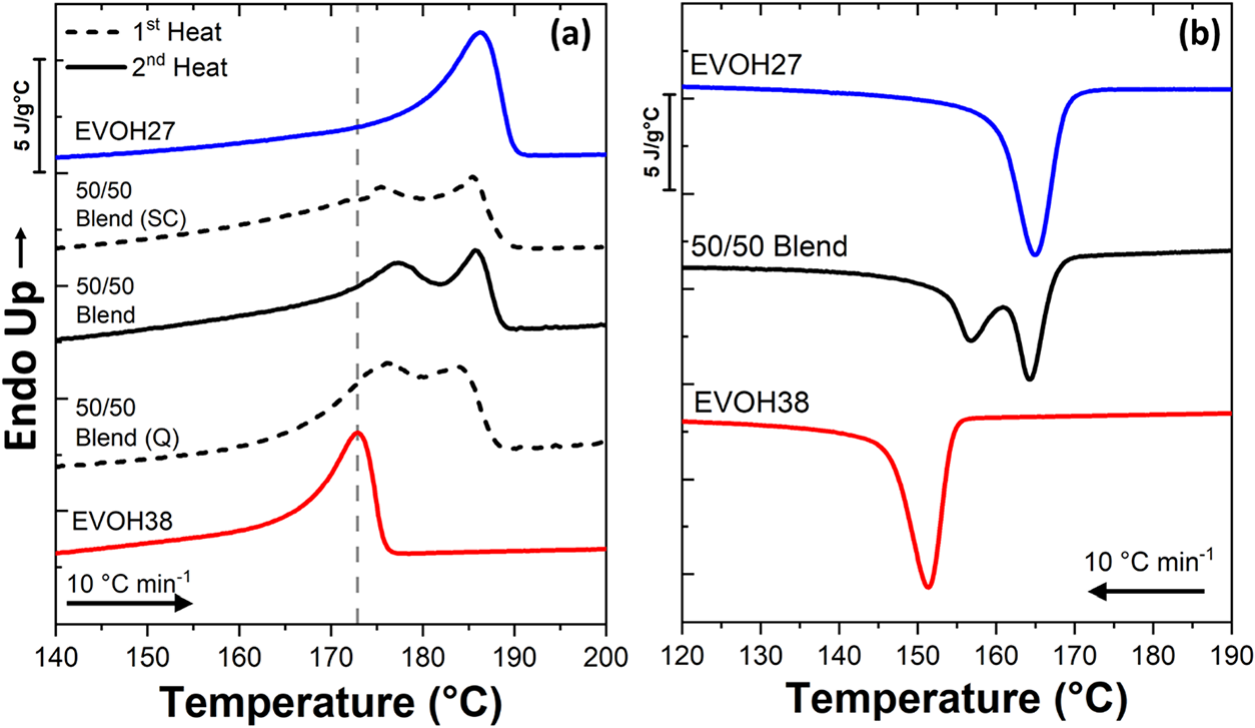
DSC thermograms of EVOH38–27 blend compared with individual components EVOH38 and EVOH27: (a) heating curves, (b) cooling curves.

Figure 7 shows DSC heating traces following isothermal crystallization at different temperatures, for two immiscible blends, EVOH48–38 (Figure 7(a)) and EVOH38–27 (Figure 7(b)). For comparison, heating traces of the higher-melting individual component for each blend, following crystallization at the same temperature, are shown as dashed curves; to facilitate a direct comparison with the 50/50 blends, the heat flow scale for the individual components is divided by 2. The short vertical bars indicate the temperatures (*T*_c_) at which the samples were isothermally crystallized. The melting behavior of EVOH38 in the EVOH48–38 blend (Δ*f*_e_ = 10 mol %, Δ*T*_f_ = 18 °C) following isothermal crystallization at higher temperatures closely resembles that of the unblended EVOH38, indicating the absence of cocrystallization in the EVOH38-rich phase under these conditions (Figure 7(a)). In contrast, at lower crystallization temperatures, partial cocrystallization of EVOH38 with EVOH48 occurred within the EVOH48-rich phase, as evidenced by the emergence of a third melting endotherm located near 165 °C, between the melting temperatures of the individual components. Nevertheless, no cocrystallization was observed in the EVOH38-rich phase, which was attributed to insufficient undercooling for EVOH48 to participate in cocrystallization, while the majority of EVOH38 crystallized rapidly. (The peak near 155 °C in all traces in Figure 7 (a) reflects crystallization of the remainder of the phase rich in EVOH48 following the isothermal crystallization, upon cooling to 10 °C.) In comparison, for blends such as EVOH38–27 (Δ*f*_e_ = 11 mol %, Δ*T*_f_ = 14 °C), isothermal crystallization at higher temperatures (*T*_c_ = 173, 172 °C), close to the melting temperature of EVOH38, resulted in melting behavior of EVOH27 in the blend that was similar to that of the neat EVOH27, indicating that nearly all of the EVOH27 crystallized independently (Figure 7(b)). However, upon decreasing the isothermal crystallization temperature (*T*_c_ = 170, 168 °C), a reduction in the melting temperature of EVOH27 in the blend was observed. This behavior reflects partial cocrystallization of EVOH27 with EVOH38 within the EVOH27-rich phase. Additionally, the presence of a shoulder near 180 °C is likely indicative of cocrystals formed between EVOH38 and EVOH27 in the EVOH38-rich phase.

**Figure 7:**
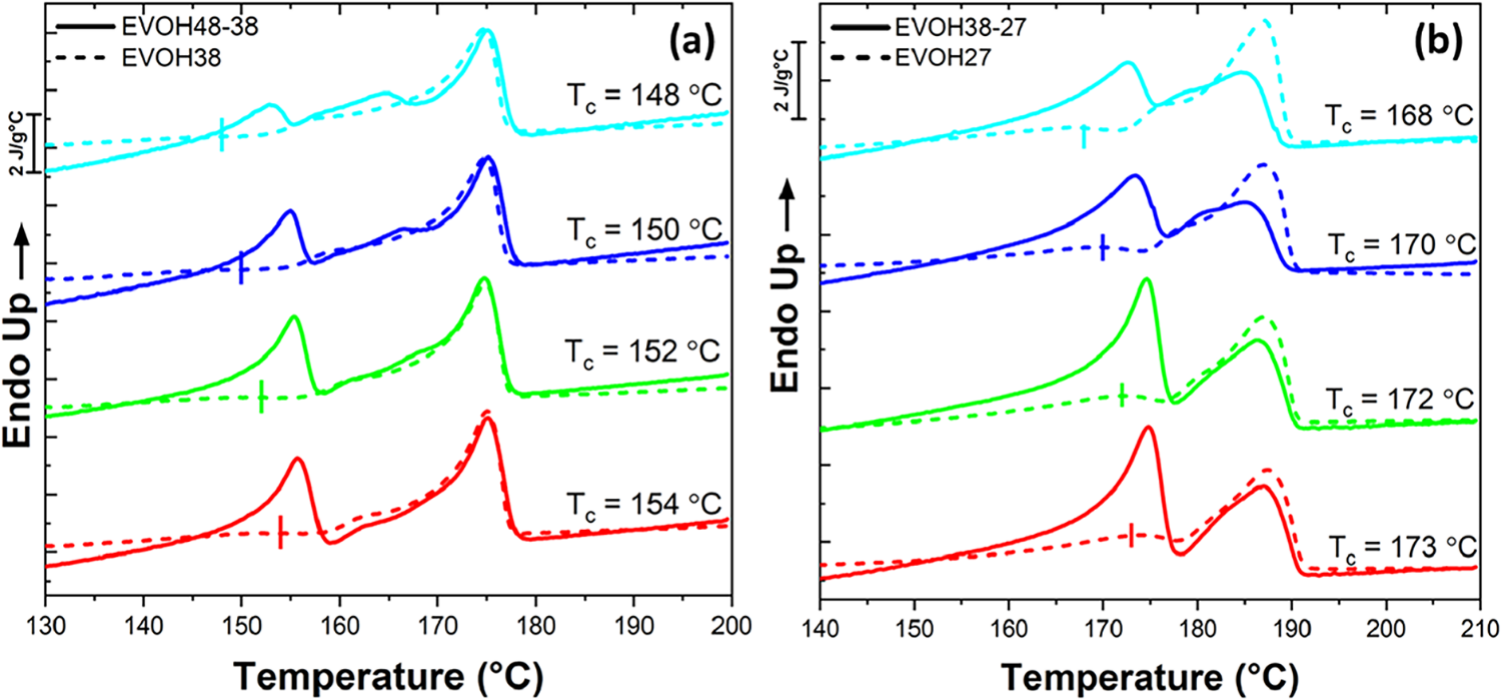
(a) Heating traces after isothermally crystallizing EVOH38 and the EVOH48–38 blend at different temperatures (*T*_c_) for 1 h and cooling at 10 °C/min to 10 °C; (b) heating traces after isothermally crystallizing EVOH27 and the EVOH38–27 blend at different temperatures and cooling at 10 °C/min to 10 °C. Short vertical bars superimposed on the dashed curves indicate the position of *T*_c_.

Generally similar results were observed for the other phase-separated blends (Figures S27–S34). For blends with Δ*T*_f_ < 15 °C, quenching brought the peak melting temperatures of the two components closer to each other (Figures 7, S33, and S34). This shows that increasing the cooling rate increased the extent of cocrystallization of the components in both phases, though the broad melting endotherm indicates the presence of multiple populations of crystals of varying composition, across both domains.

Based on our experimental findings, a miscibility and cocrystallization map was constructed for the EVOH copolymer blends (Figure 8). The upper left portion of Figure 8 shows the miscibility results, as assessed by microscopy on thin films. The lower right portion of Figure 8 shows the cocrystallization results, as assessed by thermal analysis. “Co-crystallizing” blends are those which showed a single exotherm upon cooling at 10 °C/min. Blends which exhibited two endotherms on heating, after cooling from the melt at 10 °C/min, were classified as either “partially cocrystallizing” or as “non-cocrystallizing” based on the value of *T*_m_ for the lower-temperature endotherm; if this was shifted upward by >1.5 °C from the *T*_m_ for the lower-melting individual component, the blend was classified as partially cocrystallizing, while a shift of <1.5 °C (or no shift) defined a non-cocrystallizing blend. The symmetry in Figure 8 about the 45° diagonal shows that all miscible blends cocrystallized completely upon cooling at 10 °C/min, while phase-separated blends typically showed partial cocrystallization; only in blends of copolymers with large differences in composition and *T*_f_ was cocrystallization insignificant.

**Figure 8:**
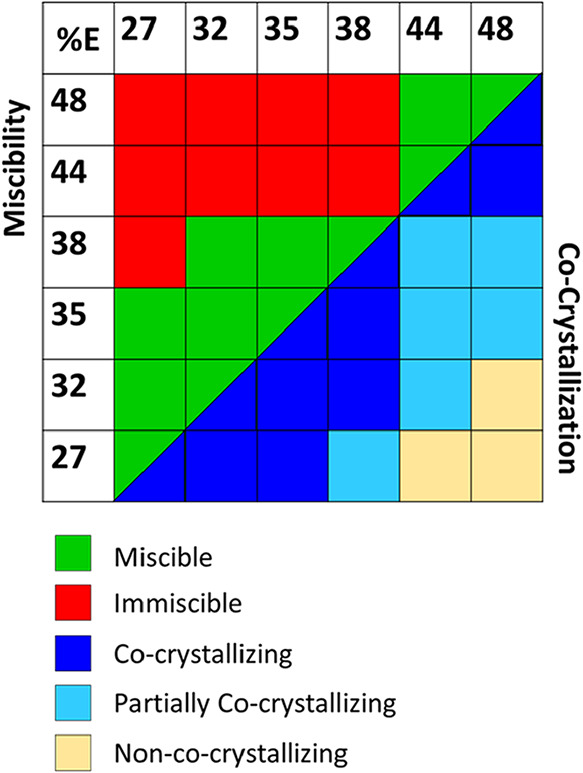
Miscibility and cocrystallization map based on experimental results.

### 3.4 Comparing Miscibility of EVOH Copolymers with Estimated Solubility Parameter Differences

With the assumption that the interaction parameter is purely enthalpic, regular solution theory gives the Flory interaction parameter χ*_AB_* in terms of the component solubility parameter difference as^30^

1 $$\chi_{\mathrm{AB}}=\frac{M}{\rho \mathrm{NRT}}{\left(\delta_{A}-\delta_{B}\right)}^{2}$$

where *M*, *N*, and ρ are the molecular weight, degree of polymerization, and density, respectively. δ_*A*_ and δ_*B*_ are the respective solubility parameters of the blend components (here, A and B are two EVOH copolymers of differing composition). Taking χ_AB_ *N* = 2 as the critical condition for miscibility, an average value of ρ = 1.03 kg m^–3^ at 220 °C for the copolymers in the studied range,^25^ and selecting a representative value of M = 90 kg mol^–1^ (discussed further below), an allowed solubility parameter difference (Δδ_c_) of 0.3 MPa^1/2^ was estimated from (eq 1). Consequently, copolymers with solubility parameter differences less than 0.3 MPa^1/2^ should be miscible.

The solubility parameter is the square root of the cohesive energy density of the material, Π_CED_. Though measurement of the heat of vaporization readily provides Π_CED_ for small-molecule liquids, this approach is not applicable to polymers. However, Π_CED_ is approximately equal to the internal pressure Π_IP_, which is accessible from pressure–volume–temperature (PVT) data; at low pressures^30^

2 $$\Pi_{\mathrm{IP}}=T\frac{\alpha }{\beta }$$

where α is the thermal expansion coefficient and β is the isothermal compressibility of the polymer. A previous study^25^ of the PVT behavior of EVOH copolymers reported values of α and β at *T* = 220 °C, which were fitted to suitable polynomials (Figures S35 and S36) in mole fraction ethylene, and those functions were used to calculate δ_IP_ ≡ Π_IP_^1/2^ via (eq 2) for any copolymer composition. As seen in Figure 9, δ_IP_ is not a linear function of composition, nor even a monotonic function. Several studies have reported such nonlinear behavior for other copolymers, in both δ_IP_ ^31^ and δ_CED_.^32–34^

**Figure 9:**
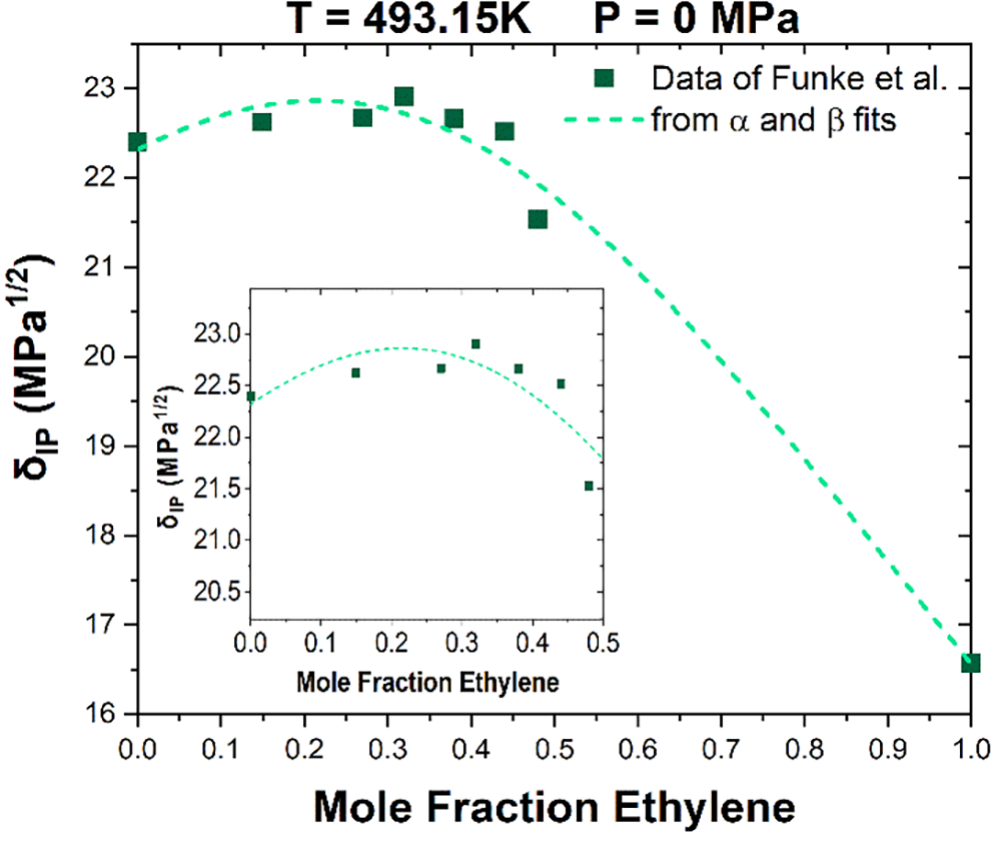
δ_IP_ for EVOH copolymers calculated from the internal pressure Π_IP_ using values of α and β measured by Funke et al.,^25^ via (eq 2). Squares represent values of δ_IP_ calculated using measured values of α and β for individual copolymers; the continuous dashed curve was calculated using polynomial functions fit to the values of α and β vs mole fraction ethylene (Figures S35 and S36).

Although the ratio $n\equiv \frac{\Pi_{\mathrm{IP}}}{\Pi_{\mathrm{CED}}}$ equals unity for a Lennard-Jones fluid, the value of *n* decreases when specific interactions are present (*n* = 0.52 for ethanol); on the other hand, *n* increases with chain length for simple hydrocarbons (*n* = 1.3 for polyethylene).^30^ A variety of polar polymers (poly­(ethylene terephthalate), nylon-66, poly­(4-vinylpyridine)) show values of *n* close to unity.^35^ Therefore, we consider *n* ≈ 1 for EVOH copolymers as well and equate Π_CED_ to Π_IP_ (δ_CED_ = δ_IP_). Blends are thus predicted to be miscible when their values of δ_*IP*_, obtained from the continuous curve in Figure 9, differ by <0.3 MPa^1/2^, the value of Δδ_c_ for *M* = 90 kg mol^–1^. These predictions are compared with experimental results in Figure 10; agreement is achieved in all cases, as evident by complete symmetry about the 45° diagonal. Of course, this result depends on the choice of *M*, as Δδ_c_ scales inversely with *M*; however, any choice of *M* ranging from 86 < *M* < 126 kg mol^–1^ would produce the same calculated diagram shown in Figure 10. Any deviations in *n* (≡Π_IP_ /Π_CED_) from unity would have only a modest impact on the value of *M* needed to match experiment, such that *n* ^1/2^ *M* = 90 kg mol^–1^. For blends of polydisperse polymers adequately described by a common Schulz–Flory distribution, the critical point is determined by the weight-average molecular weight, *M*_w_.^36^ Commercial EVOH copolymers of generally similar melt index to the polymers studied here have been reported to have *M*_w_ values ranging from 81–86 kg mol^–1^, as measured by gel permeation chromatography.^37,38^

**Figure 10:**
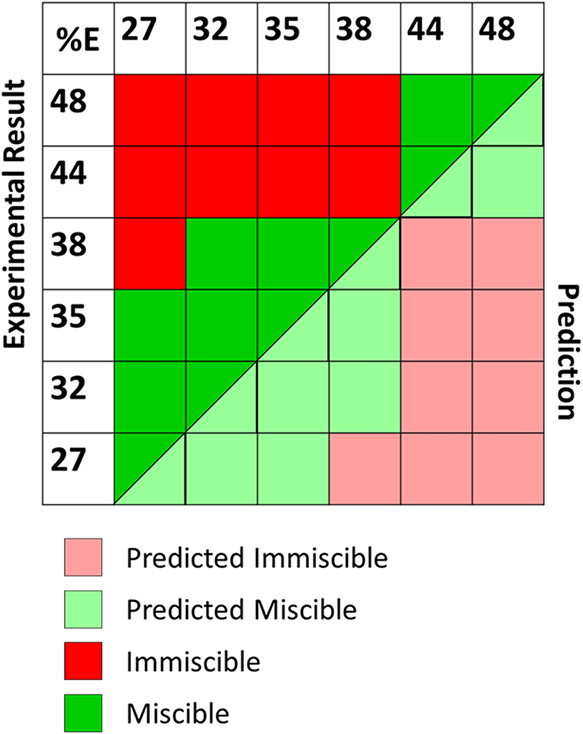
Experimental results for miscibility compared with predictions of regular mixing with *M* = 90 kg mol^–1^.

The nonlinearity in δ_IP_ implies that the critical composition difference for miscibility does not correspond to a fixed percent ethylene, but that this value should change with the average comonomer content in the blend. This feature is evident in Figure 10: EVOH38–32 (Δ*f*_e_ = 6 mol %) is miscible, whereas EVOH44–38 (Δ*f*_e_ = 6 mol %) showed phase separation. Also, as mentioned previously (Figure 4), the domain size in EVOH38–27 (Δ*f*_e_ = 11 mol %) is smaller than in EVOH44–35, implying a smaller interfacial tension (which also increases with Δδ), even though the difference in composition is greater. All the EVOH copolymers studied here have ethylene contents beyond the maximum in δ_IP_ vs *f*_e_; the downward curvature in δ_IP_ indicates that as the average ethylene content in the blend is increased, it becomes more difficult for the copolymers to form miscible blends.

Although the miscibility prediction based on δ_*IP*_ works well for the EVOH copolymers, it is not very accurate when extended to PVOH. As shown in Figure 9, δ_IP_ = 22.32 MPa^1/2^ for PVOH; because δ_IP_ shows a maximum as a function of ethylene fraction, this same value of δ_IP_ = 22.32 MPa^1/2^ is also found for a copolymer with 41.7 mol % ethylene, and this value is within the critical difference for miscibility (0.3 MPa^1/2^) relative to the values of δ_IP_ for EVOH38 and EVOH44. Based purely on δ_IP_, PVOH is thus predicted to be miscible with both EVOH38 and EVOH44. However, PVOH is found experimentally to be immiscible with not only these two copolymers, but also with EVOH35, EVOH32, and EVOH27 (Figures S37–S41). A likely explanation for this disagreement is that *n* (≡Π_IP_ /Π_CED_) varies with composition, since *n* is decreased by hydrogen bonding between VOH units. If *n* increases with ethylene content, this will attenuate (or even eliminate) the predicted maximum in δ_CED_ when compared with the maximum in δ_IP_, shifting the predicted miscibility window for EVOH copolymers with PVOH.

## 4 Conclusions

The miscibility and cocrystallization behaviors of EVOH copolymer blends with varying ethylene content (27 – 48 mol % ethylene) were systematically investigated. An analog of the solubility parameter, δ_IP_ calculated from literature PVT data, showed a nonlinear dependence on ethylene content, indicating that the critical composition difference for miscibility is predicted to depend on the average ethylene content in the blend. The experimentally observed blend miscibility matched the predictions of regular mixing, using these values of δ_IP_ and *M* = 90 kg mol^–1^. Miscible blends generally behaved as single copolymers of intermediate composition, undergoing complete cocrystallization at moderate cooling rates. The extent of cocrystallization was governed primarily by the difference in freezing temperatures (Δ*T*_f_) and the cooling rate. When Δ*T*_f_ > 7 °C, complete cocrystallization was not achieved in miscible blends at slower cooling rates, which yielded only partial cocrystallization. In contrast, blends with larger composition differences showed phase-separated domains, accompanied by surface roughening in thin films due to preferential wetting of the blend components at the surface and substrate. Partial cocrystallization was also observed in several immiscible blends, indicating that the coexisting melt phases were not pure components. In immiscible blends with Δ*T*_f_ < 15 °C, the melting peaks shifted toward each other under rapid cooling, indicating enhanced cocrystallization in both domains. Overall, the ability of EVOH copolymers to partially cocrystallize even in phase-separated systems suggests that cocrystallization should also occur at the domain interfaces, enhancing mechanical compatibility and supporting the potential recyclability of EVOH copolymers together in a single stream.

## Supplementary Material



## Notes

Effect of extrusion process on EVOH copolymers; list of processing conditions; list of blends prepared; OM images; AFM height images; 1D autocorrelation function plots; DSC thermograms; X-ray diffraction patterns; polynomial fits of α and β; and OM images of EVOH-PVOH blends (PDF)
