# Supplementary material for: Miscibility and Cocrystallization in Ethylene–Vinyl Alcohol Copolymer Blends
Source: ACS Appl Polym Mater. 2026 Jun 9;8(12):9991–10000. doi: 10.1021/acsapm.6c01860 (PMC13316853; doi:10.1021/acsapm.6c01860)
Supplement: Supplementary file 1 [file ap6c01860_si_001.pdf]

Supporting Information:

Miscibility and Cocrystallization in Ethylene-Vinyl Alcohol Copolymer Blends

*Asmita Ghosh, Richard A. Register**

Department of Chemical and Biological Engineering, Princeton University, NJ 08544

*register@princeton.edu

Thermal Properties of Untreated and Extruded EVOH Copolymers

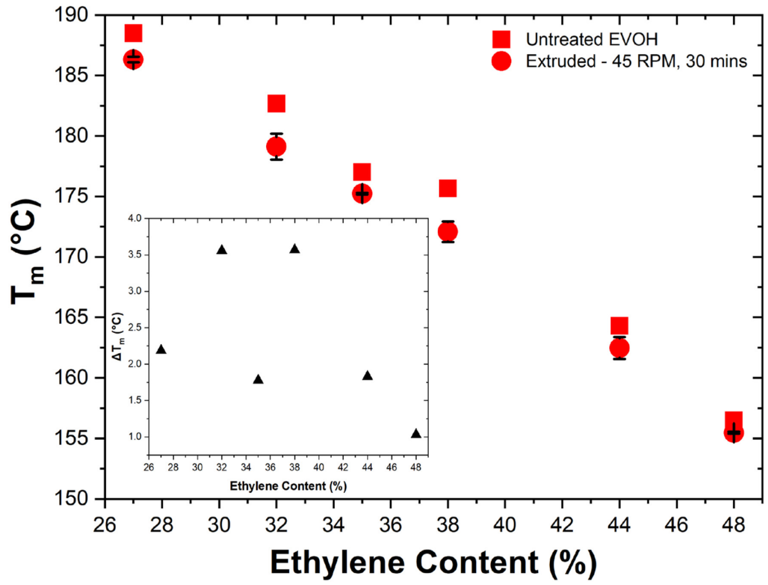

Figure S1. Effect of extrusion process on T_m of individual EVOH copolymers.

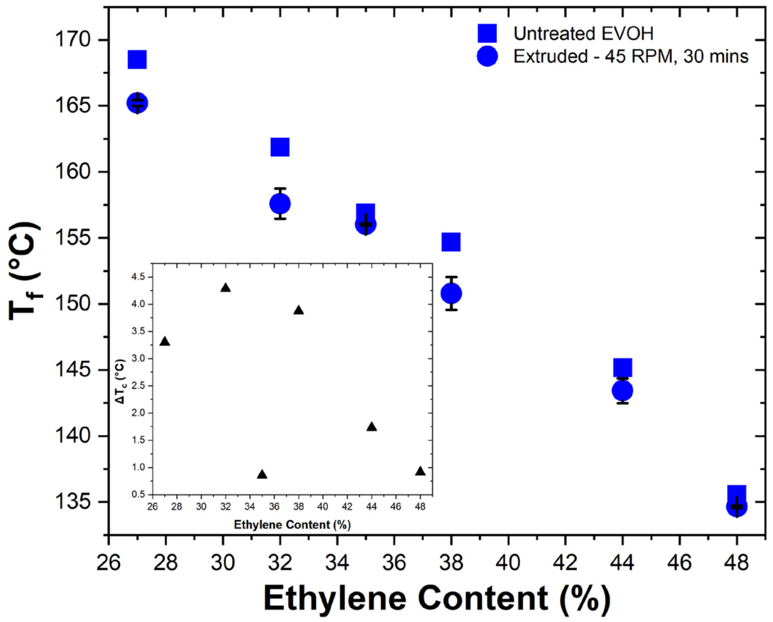

Figure S2. Effect of extrusion process on T_f of individual EVOH copolymers.

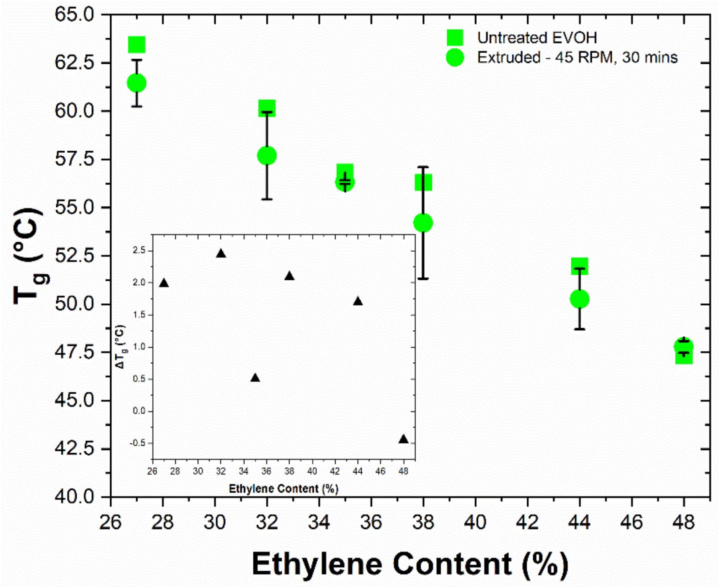

Figure S3. Effect of extrusion process on T_g of individual EVOH copolymers.

Extrusion of Individual EVOH Copolymers

Table S1. Processing temperatures of extruded individual copolymers

Copolymer	Processing Temperatures (°C)
EVOH48	210
EVOH44	190
EVOH38[a]	200, 190
EVOH35	200
EVOH32[a]	210, 200
EVOH27	210

[a]Material was processed at either of the two indicated temperatures, differing by 10 °C; no discernible difference in behavior was observed.

Table S2. List of blends studied in this work

Blend	Difference in Ethylene Content (Δf_e) (mol %)	Average Ethylene Content (mol %)	Processing Temperature (°C)
EVOH48-27	21	38.0	210
EVOH48-32	16	40.3	200
EVOH48-35	13	41.7	200
EVOH48-38	10	43.1	190
EVOH48-44	4	46.0	190
EVOH44-27	17	35.8	210
EVOH44-32	12	38.1	200
EVOH44-35	9	39.6	200
EVOH44-38	6	41.0	190
EVOH38-27	11	32.6	210
EVOH38-32	6	35.0	200
EVOH38-35	3	36.5	200
EVOH35-27	8	31.1	210
EVOH35-32	3	33.5	200
EVOH32-27	5	29.5	210

Optical Microscopy Images

a) EVOH Blends

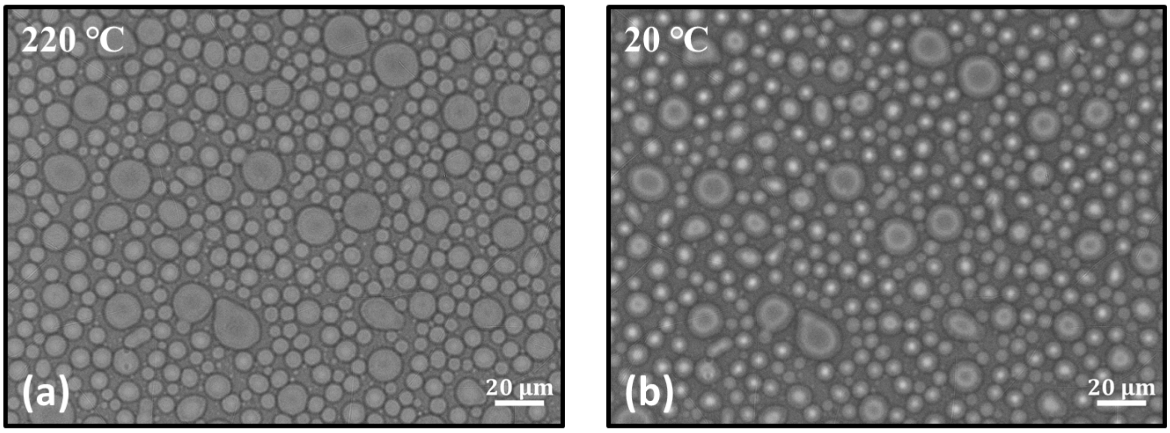

Figure S4. OM image showing EVOH48-32 blend as seen in the melt (a) and after cooling (b).

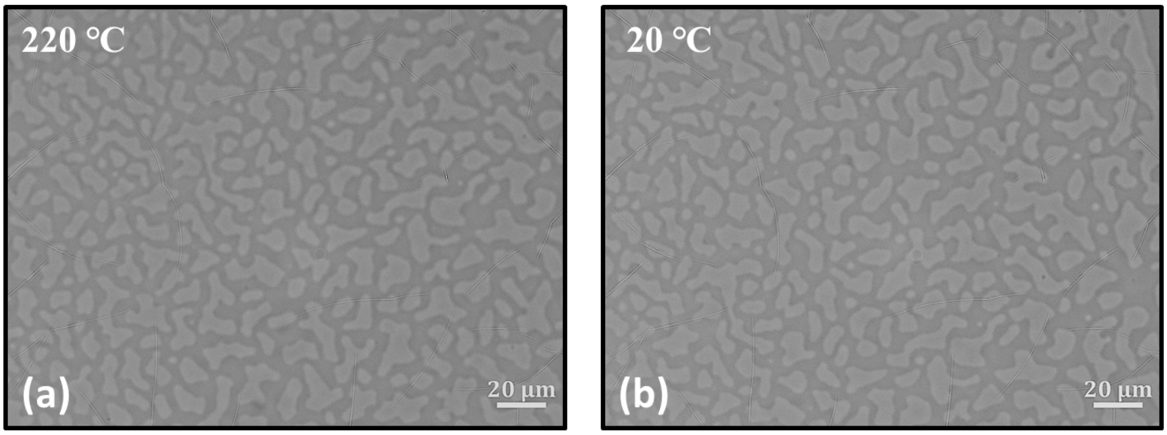

Figure S5. OM image showing EVOH48-35 blend as seen in the melt (a) and after cooling (b).

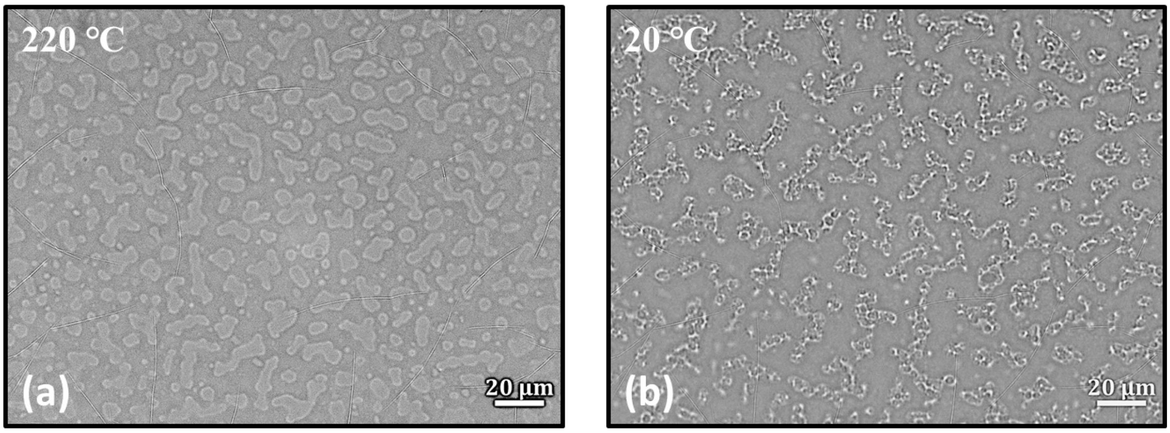

Figure S6. OM image showing EVOH48-38 blend as seen in the melt (a) and after cooling (b).

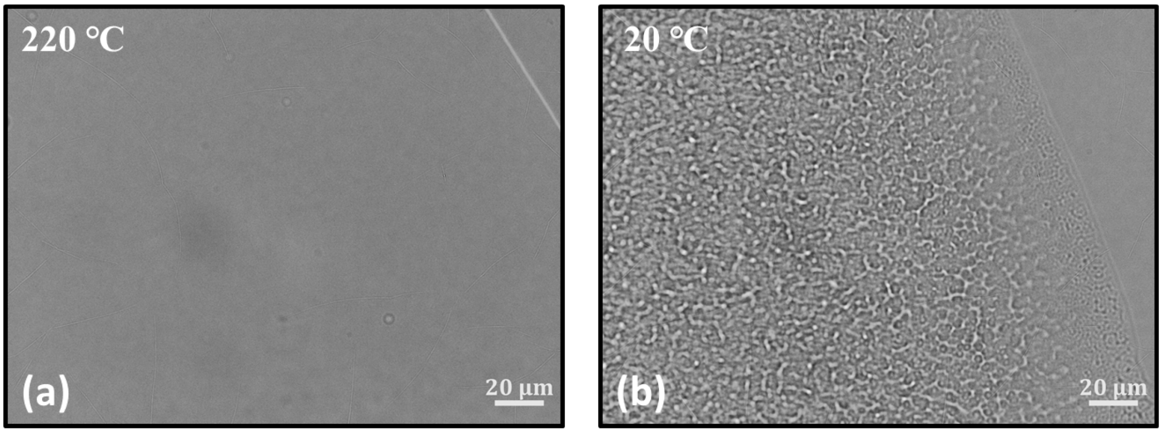

Figure S7. OM image showing EVOH48-44 blend as seen in the melt (a) and after cooling (b).

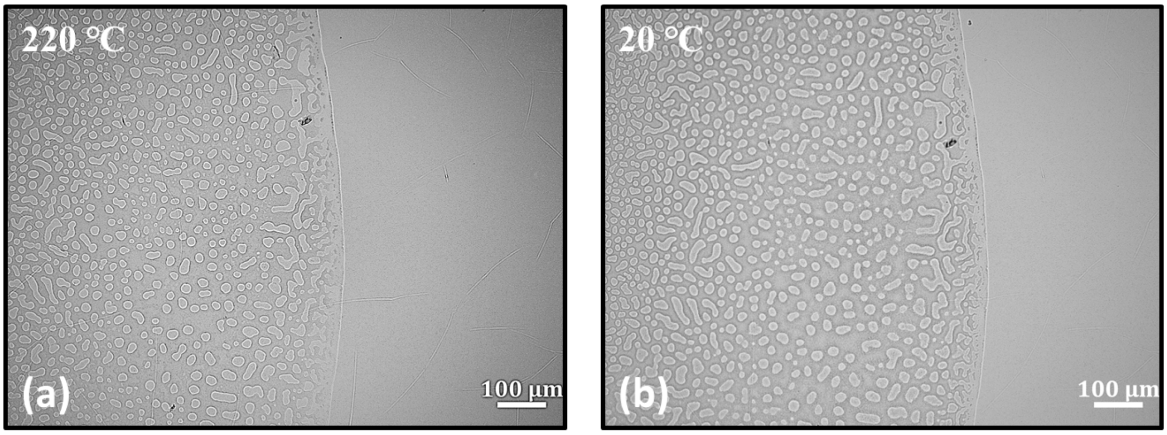

Figure S8. OM image showing EVOH44-27 blend as seen in the melt (a) and after cooling (b).

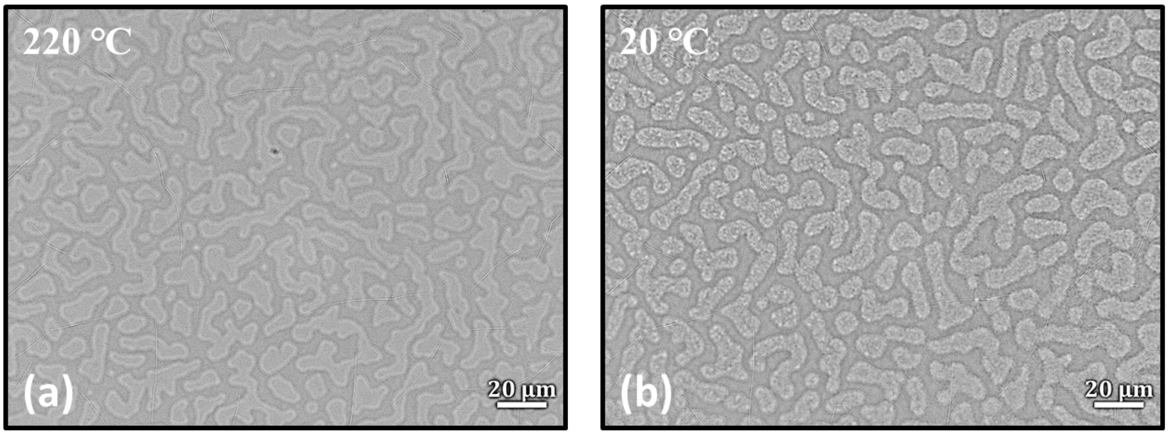

Figure S9. OM image showing EVOH44-32 blend as seen in the melt (a) and after cooling (b).

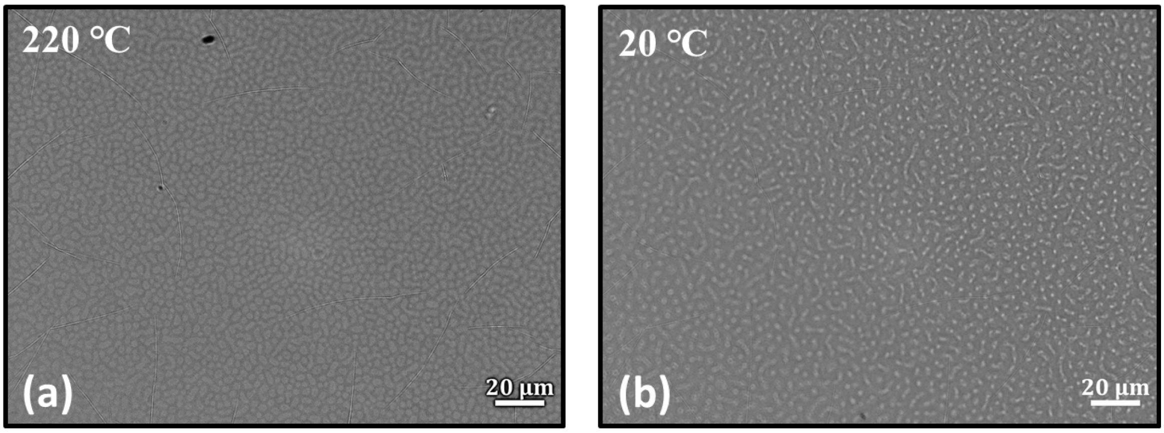

Figure S10. OM image showing EVOH44-35 blend as seen in the melt (a) and after cooling (b).

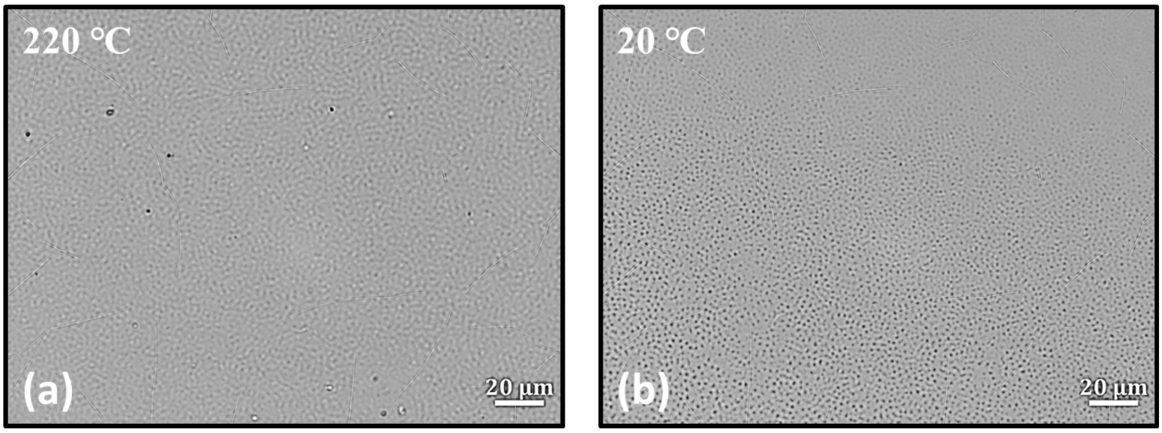

Figure S11. OM image showing EVOH44-38 blend as seen in the melt (a) and after cooling (b).

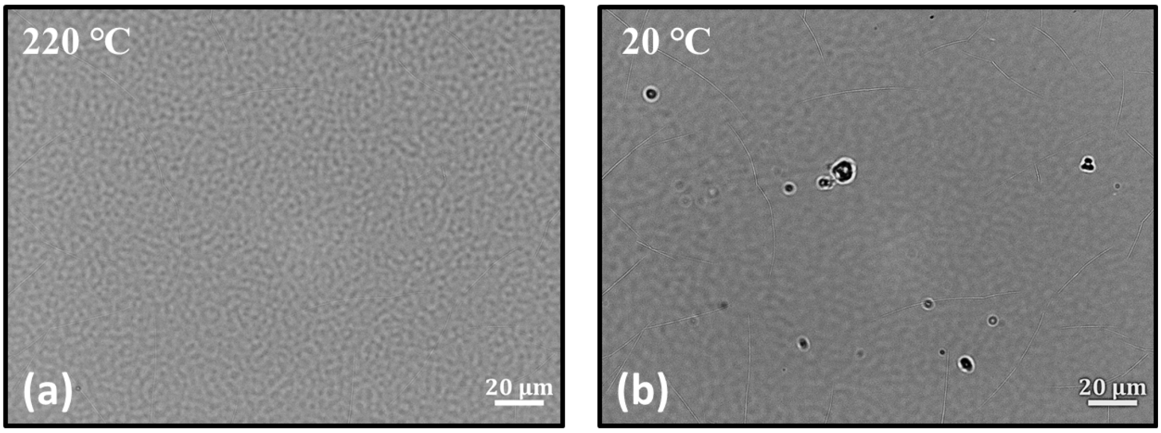

Figure S12. OM image showing EVOH38-27 blend as seen in the melt (a) and after cooling (b).

b) Individual Components

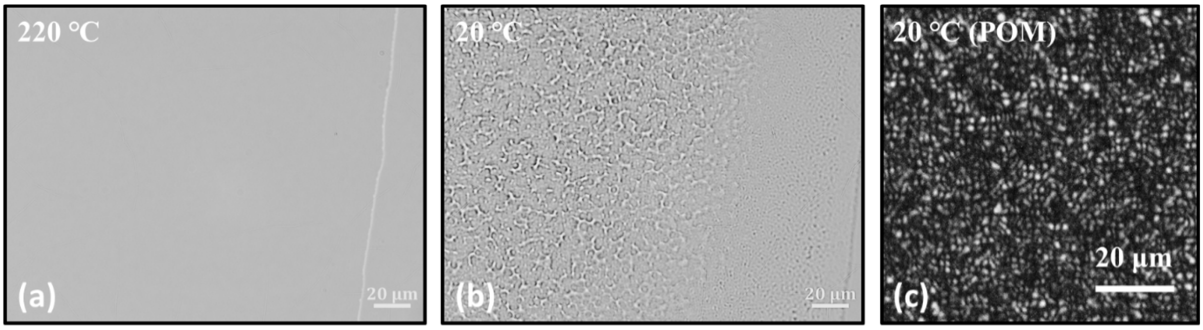

Figure S13. OM image showing EVOH35 as seen in the melt (a), after cooling (b), and under POM (80 µm × 80 µm) (c). EVOH35 upon melting produces a homogeneous film without any signs of film rupture. POM at room temperature shows formation of crystal superstructures.

Evolution of domains with time in phase separated blends

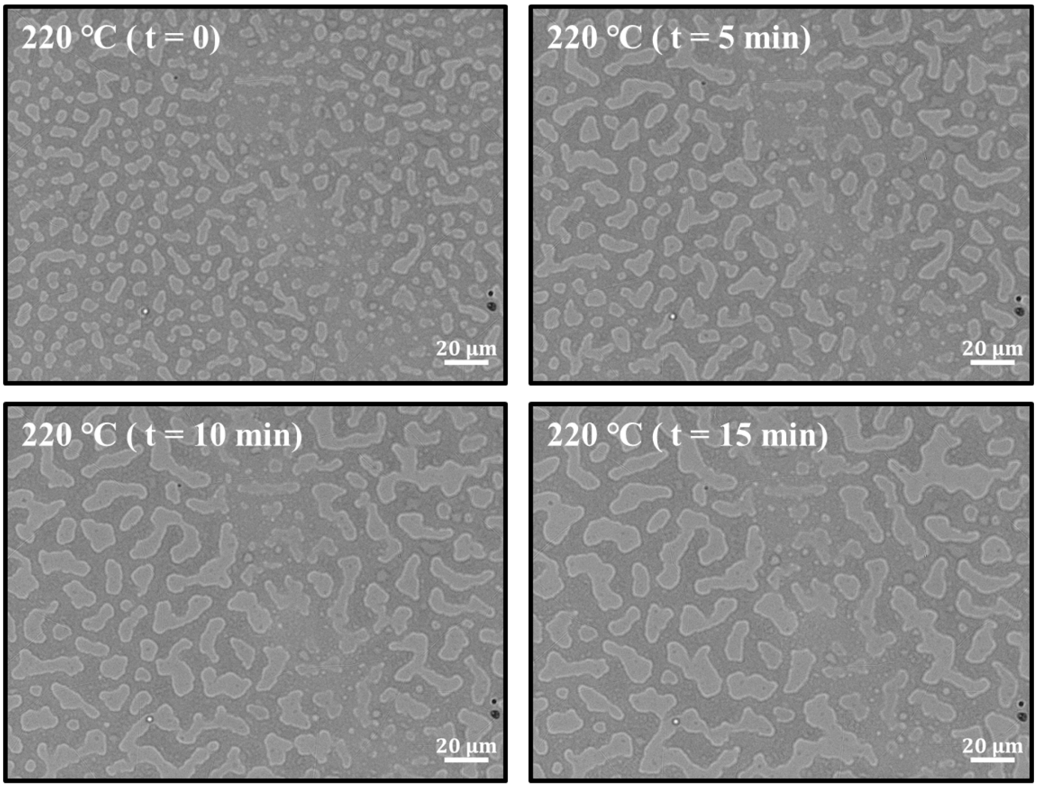

Figure S14. Coarsening of domains in EVOH48-27 blend as observed under bright field optical microscope.

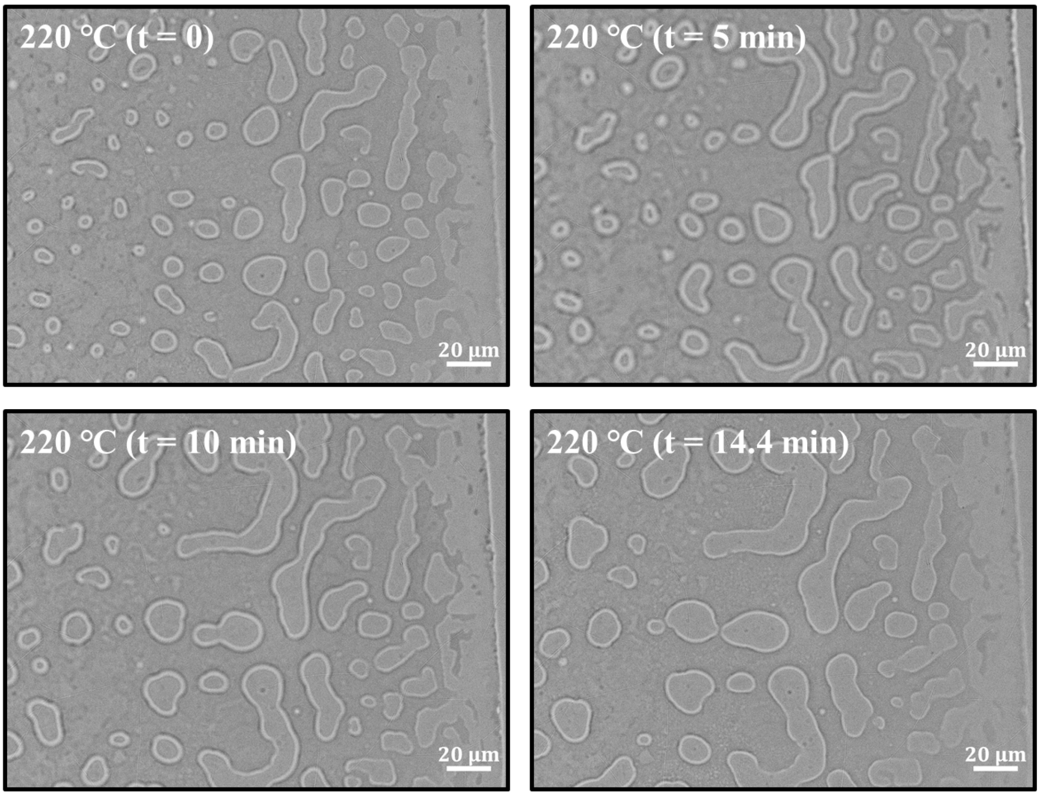

Figure S15. Coarsening of domains in EVOH44-27 blend as observed under bright field optical microscope.

AFM Images

a) Individual Components

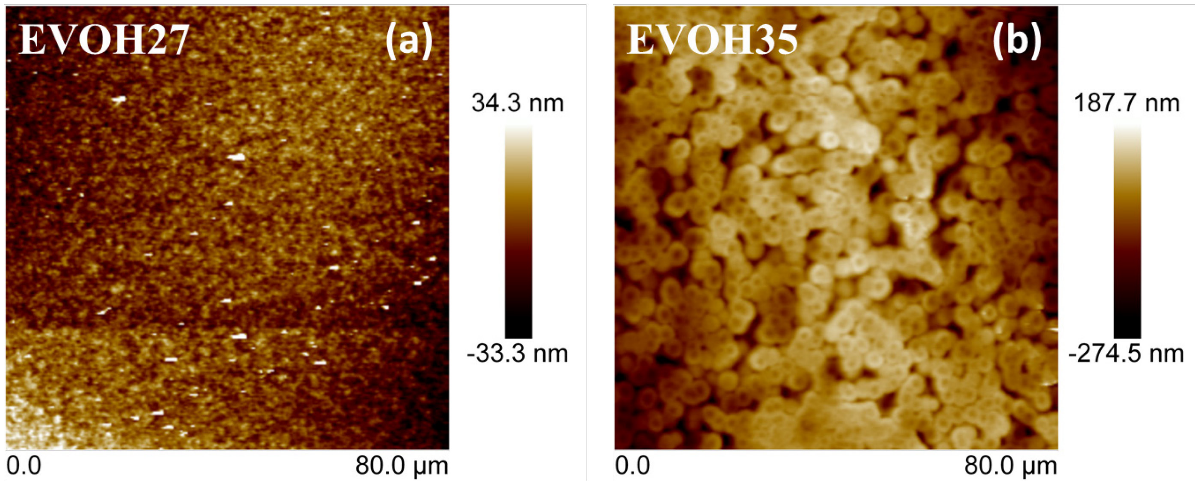

Figure S16. AFM height images of EVOH27 (a) and EVOH35 (b) individual component films. The circular structures observed in EVOH35 (panel (b)) show the formation of spherulites, as also observed under POM in **Fig S13 (c)**.

b) Miscible Blends

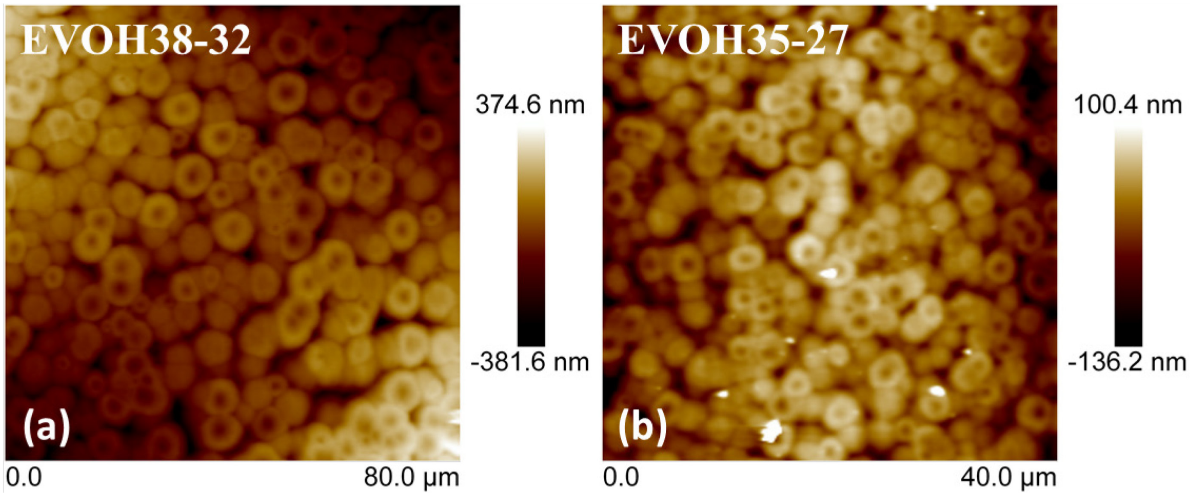

Figure S17. AFM height images of miscible EVOH blend films: (a) EVOH38-32; (b) EVOH35-27.

Immiscible Blends

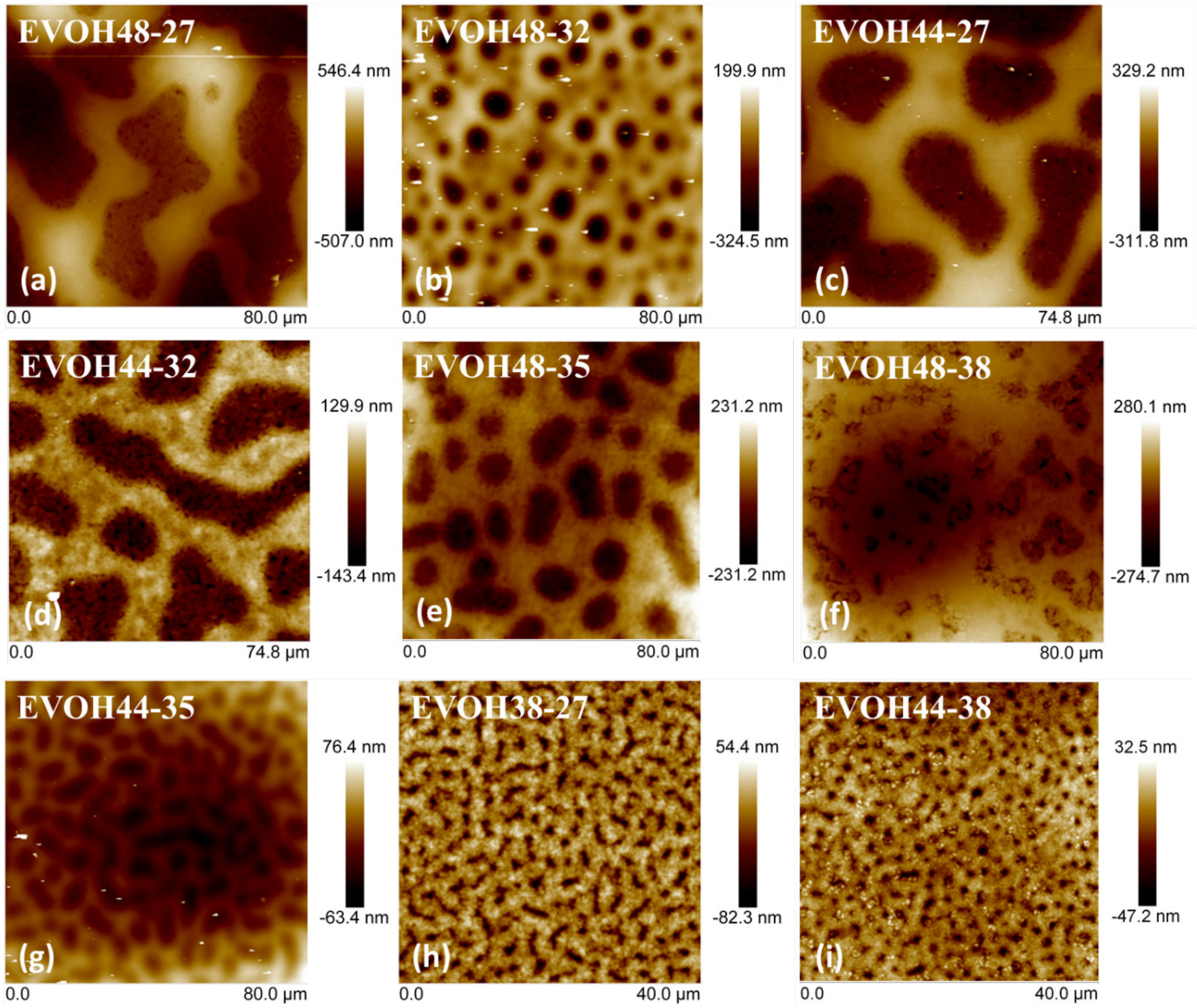

Figure S18. AFM height images of phase-separated EVOH blend films: (a) EVOH48-27; (b) EVOH48-32; (c) EVOH44-27; (d) EVOH44-32; (e) EVOH48-35; (f) EVOH48-38; (g) EVOH44-35; (h) EVOH38-27; (i) EVOH44-38.

Comparing AFM and Optical Microscopy Results

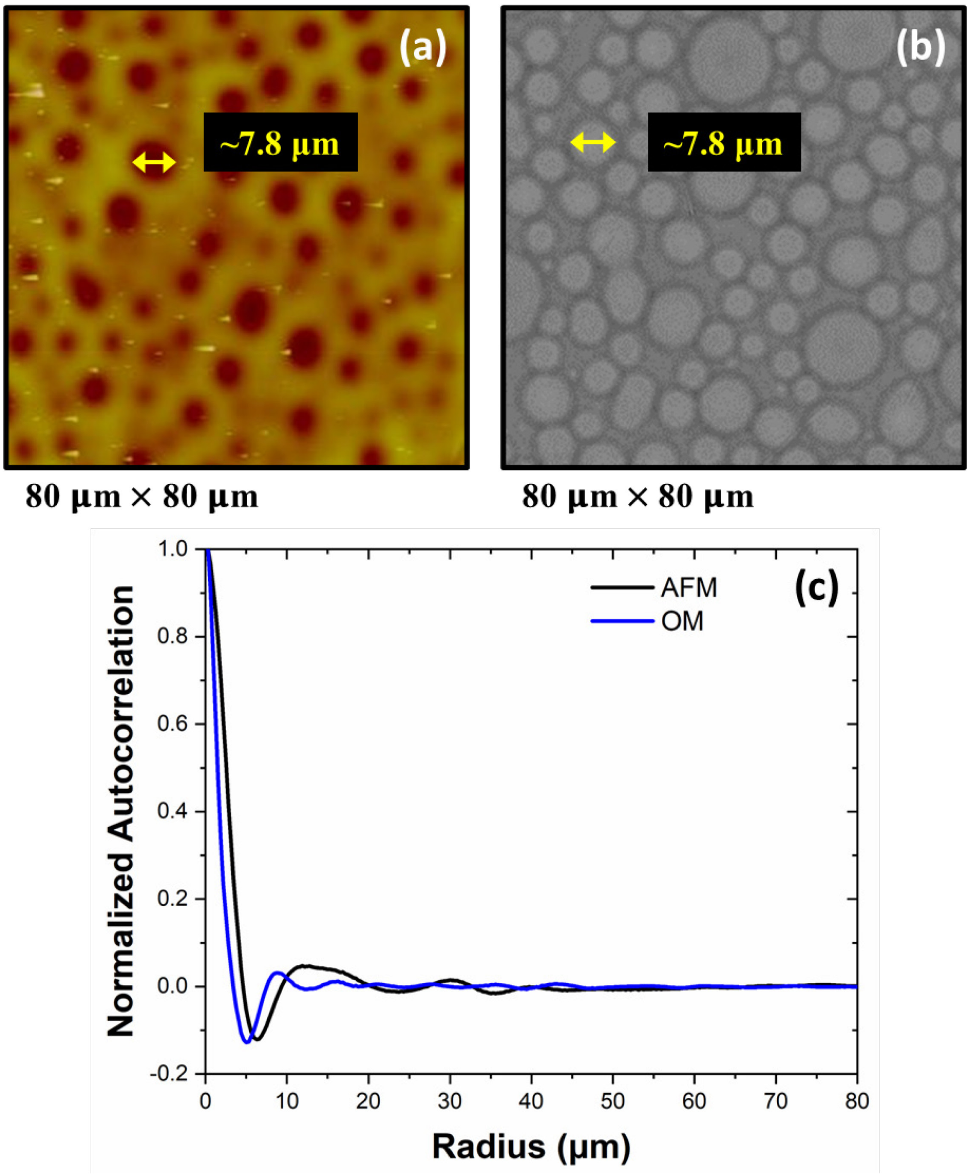

Figure S19. (a) AFM height image of EVOH48-32 blend; (b) 80 µm × 80 µm snippet (scaled to 256 pixels) taken from the OM image of molten EVOH48-32 blend; (c) 1D autocorrelation functions computed from the images in (a) and (b). The double-headed arrow indicates the length of a particular feature depicted in each of the images, which do not correspond to the same area of the film.

The correlation lengths obtained from the AFM and OM images are similar. This shows that the features observed by both AFM and optical microscopy are of similar length scale and occur due to phase separation in the blends, leading to surface roughening of the film.

Correlation Functions for Phase-separated Blend Films

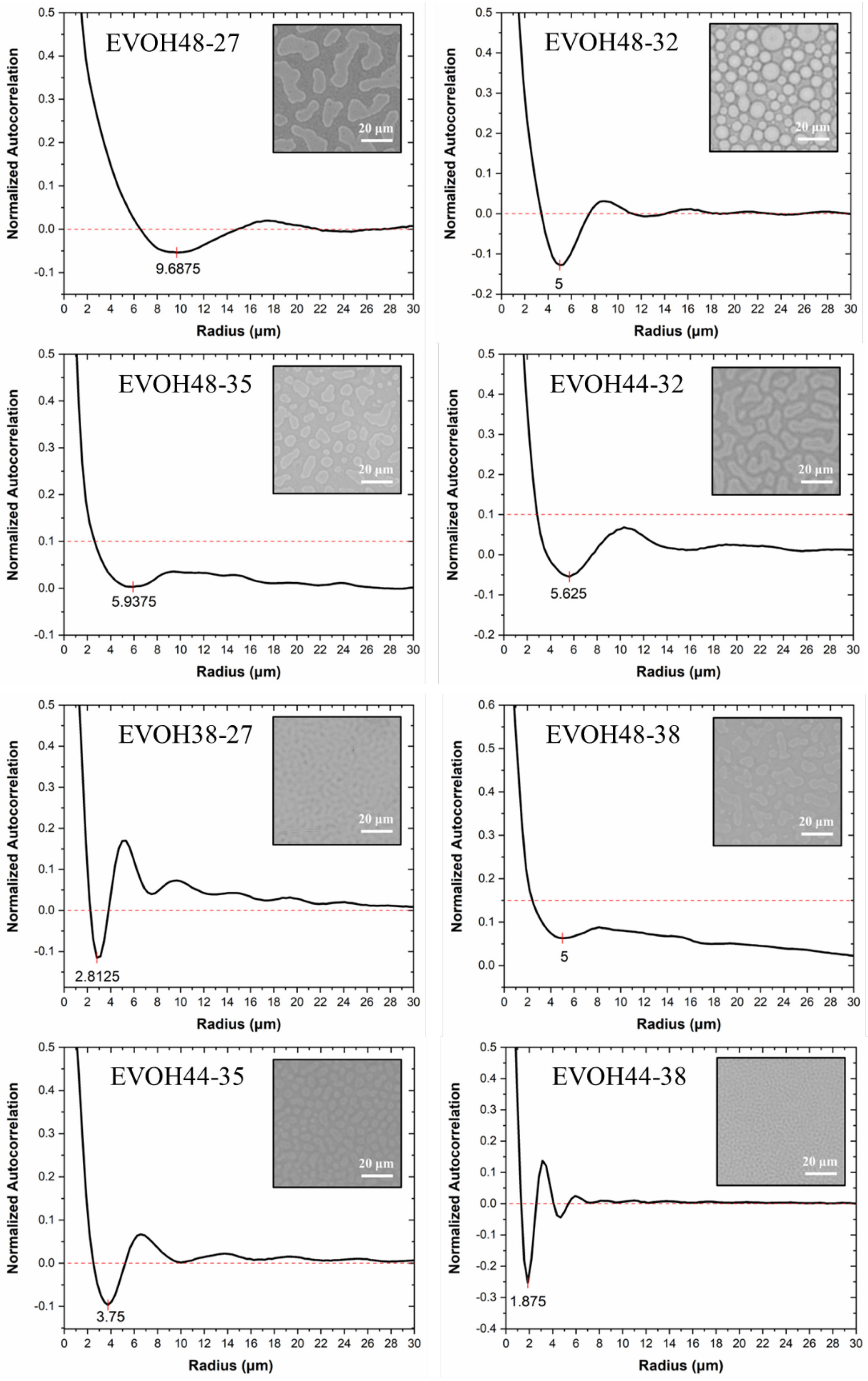

Figure S20. Normalized 1D autocorrelation functions of phase-separated EVOH blends as indicated. Insets show the OM images of the corresponding blend at 220 °C, held for 15 mins or longer.

Thermal and X-Ray Diffraction Analysis

a) Miscible and completely co-crystallizing blends under all conditions

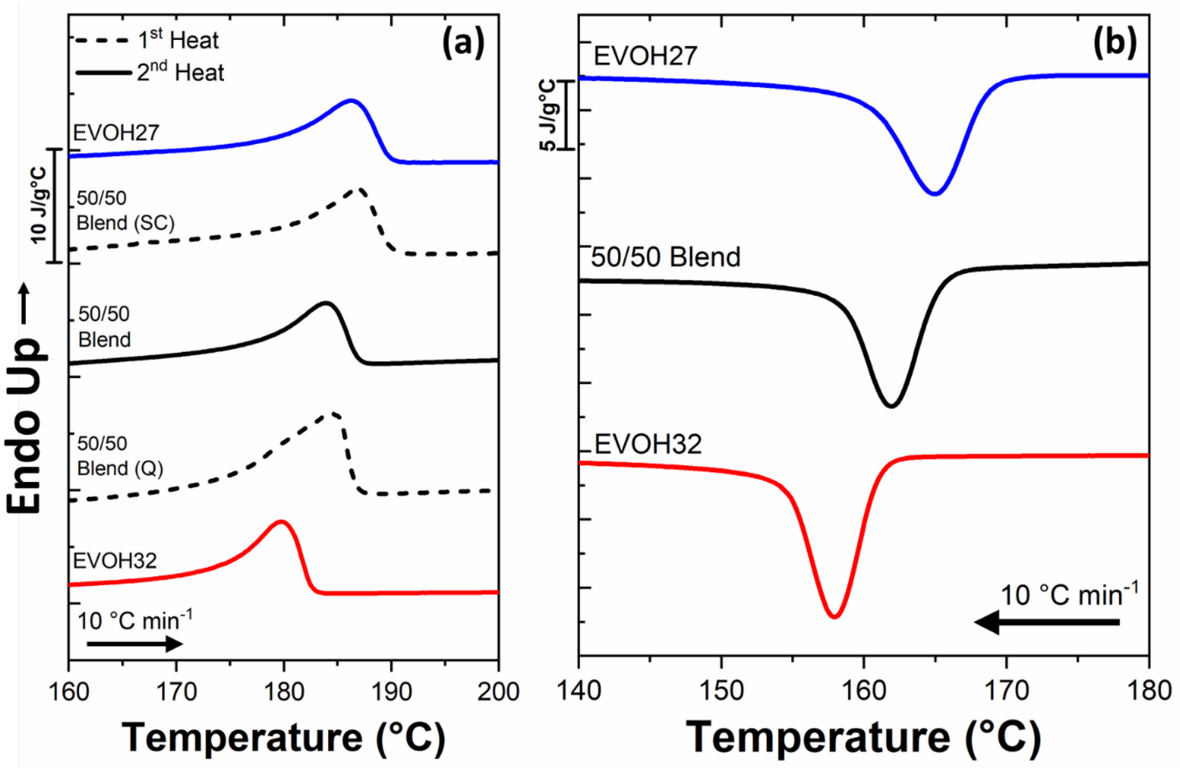

Figure S21. DSC thermograms of EVOH32-27 blend compared with individual components processed at 210 °C: (a) heating curves (b) cooling curves.

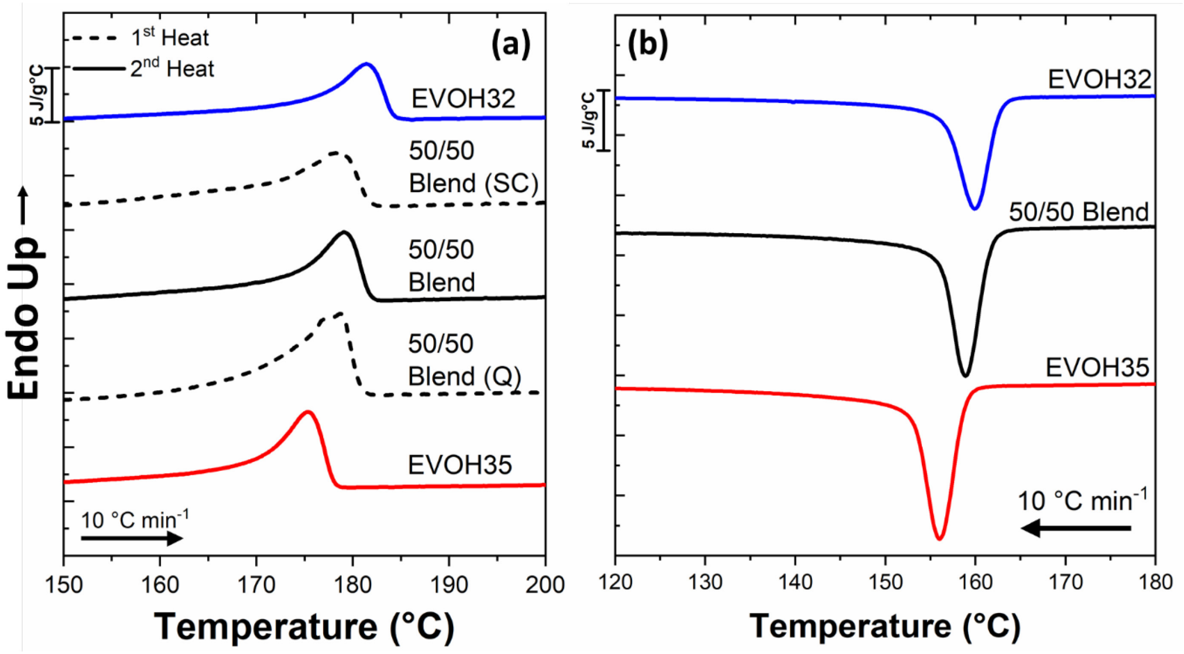

Figure S22. DSC thermograms of EVOH35-32 blend compared with individual components processed at 200 °C: (a) heating curves (b) cooling curves.

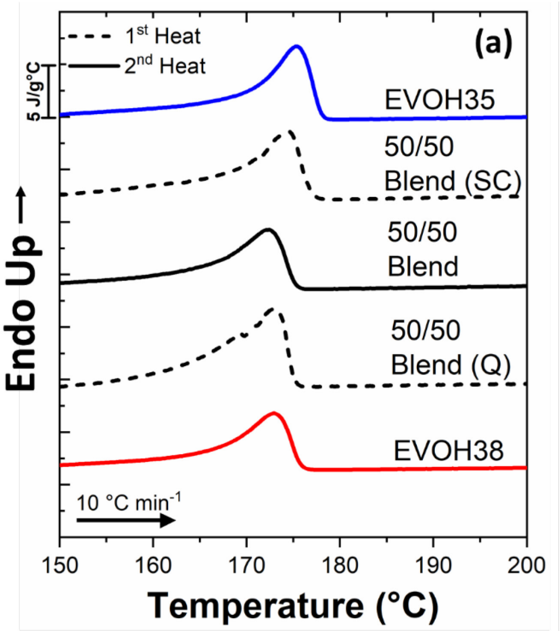

Figure S23. DSC thermograms of EVOH38-35 blend compared with individual components processed at 200 °C: (a) heating curves (b) cooling curves.

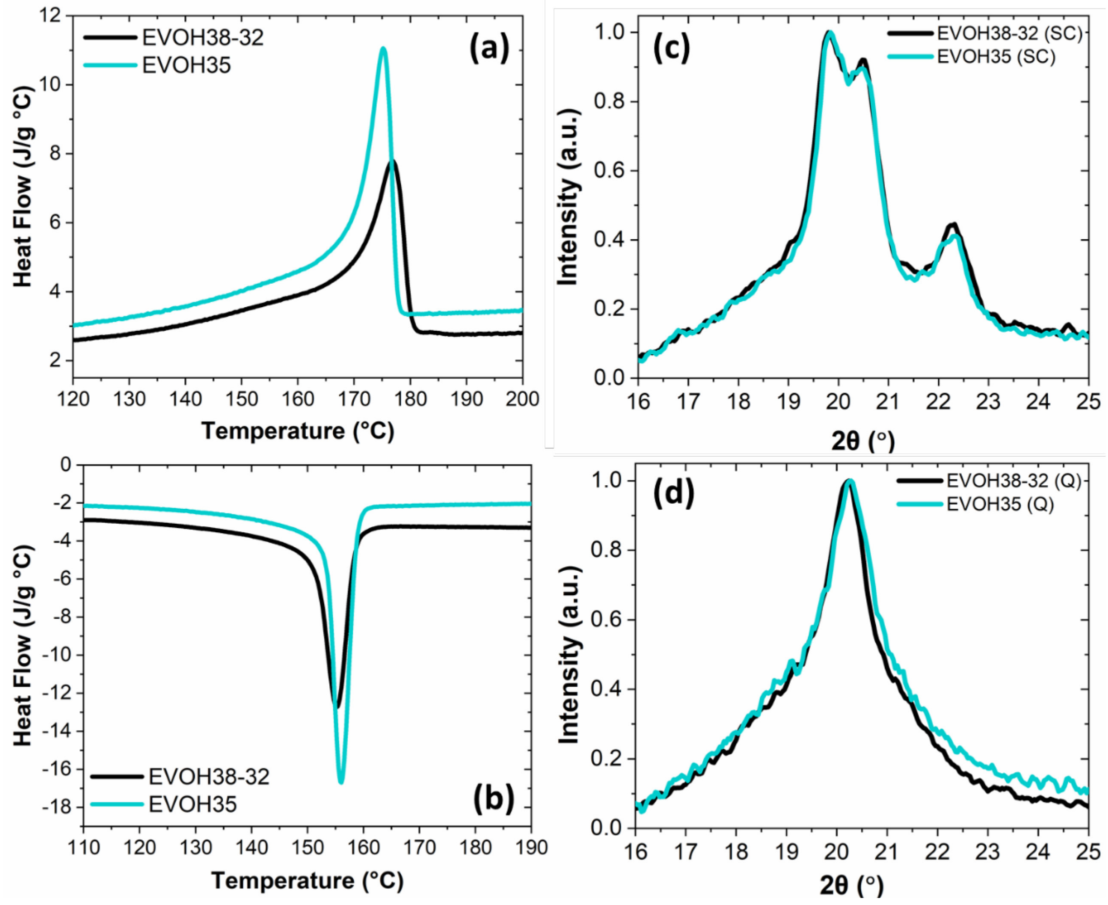

Figure S24. DSC thermograms of EVOH38-32 blend compared with EVOH35 processed under similar conditions: (a) heating trace; (b) cooling trace. Heating and cooling rate is 10 °C/min. XRD pattern of slowly cooled (c) and quenched (d) samples of EVOH38-32 and EVOH35 processed under similar conditions.

b) **Miscible and partially co-crystallizing blends under certain conditions**

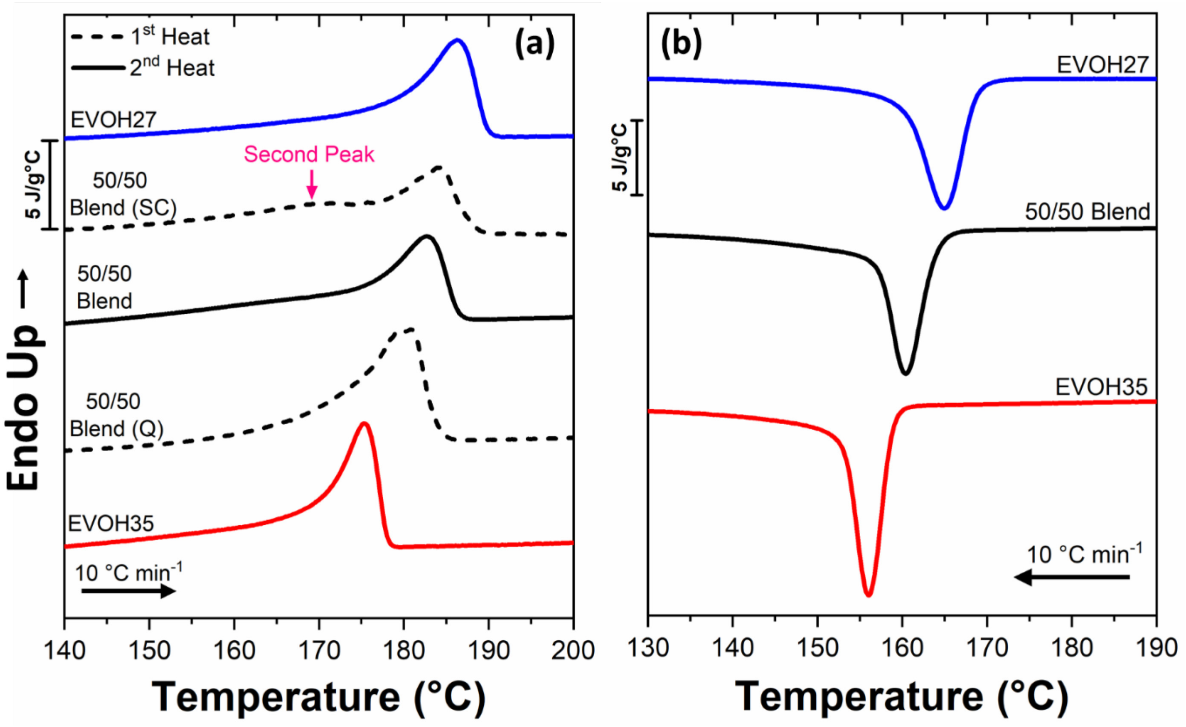

Figure S25. DSC thermograms of EVOH35-27 blend compared with individual components processed at 200 °C (EVOH35) and 210 °C (EVOH27): (a) heating curves (b) cooling curves.

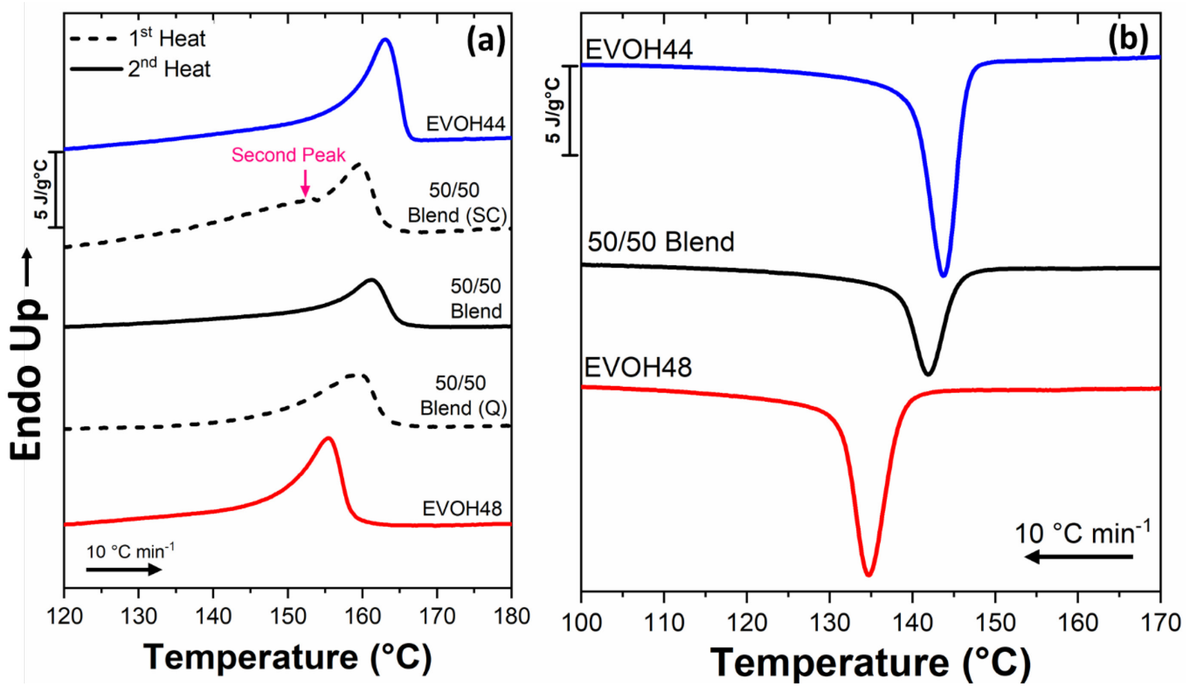

Figure S26. DSC thermograms of EVOH48-44 blend compared with individual components processed at 190 °C (EVOH44) and 210 °C (EVOH48): (a) heating curves (b) cooling curves.

c) Phase-separated blends

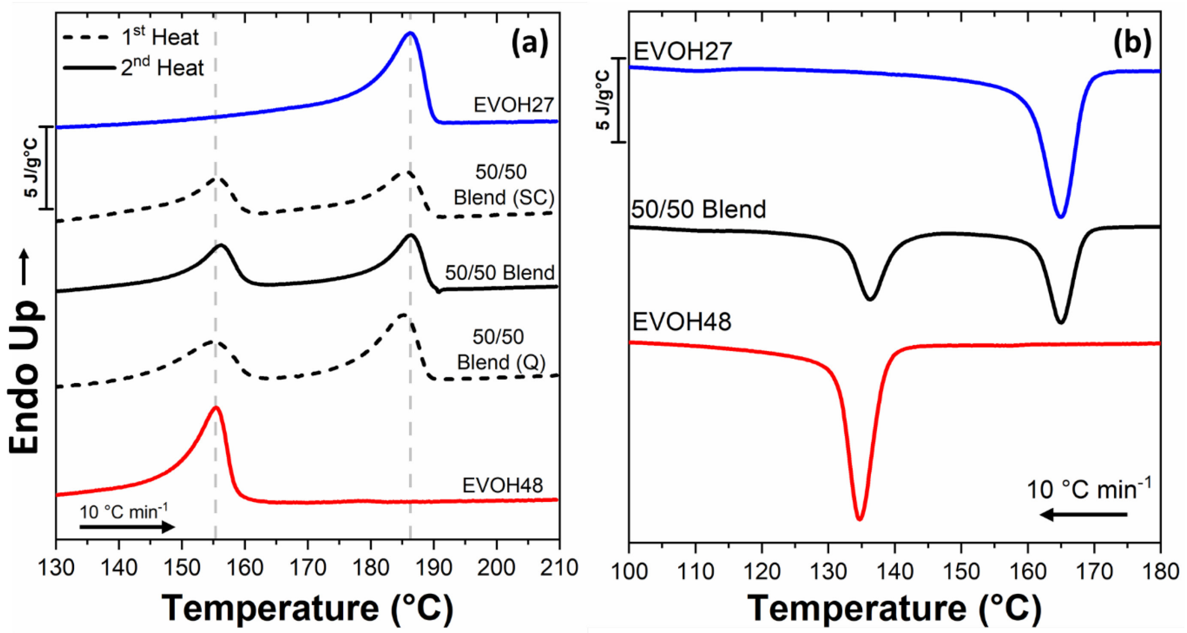

Figure S27. DSC thermograms of EVOH48-27 blend compared with individual components processed at 210 °C: (a) heating curves (b) cooling curves.

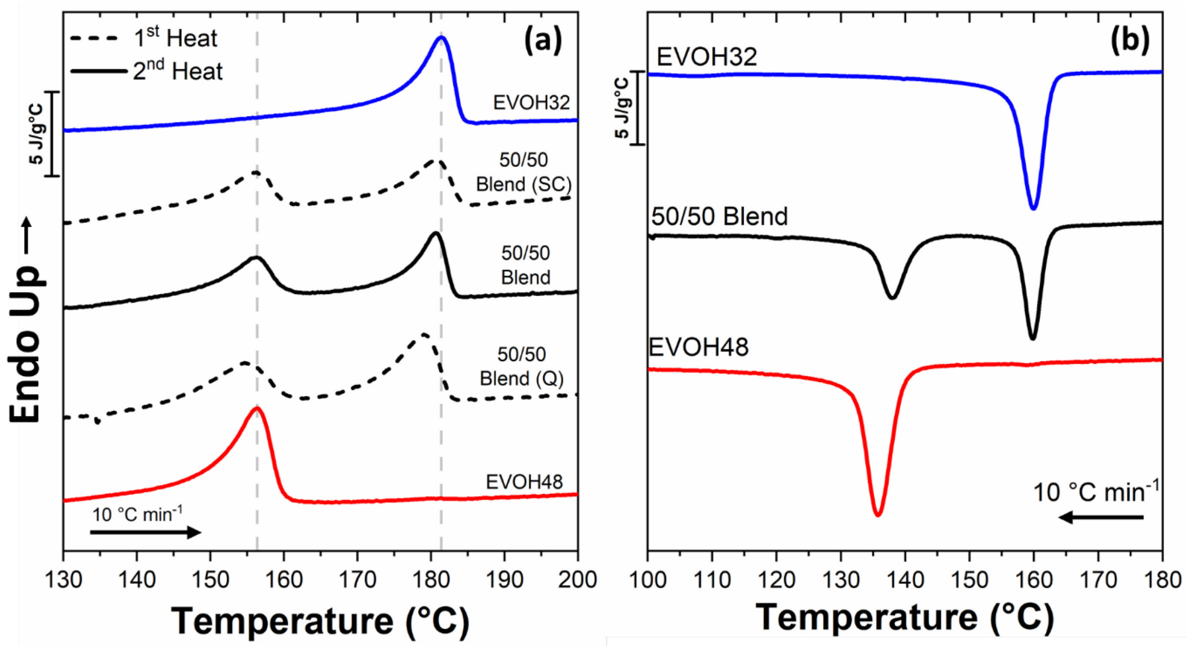

Figure S28. DSC thermograms of EVOH48-32 blend compared with individual components processed at 210 °C (EVOH48) and 200 °C (EVOH32): (a) heating curves (b) cooling curves.

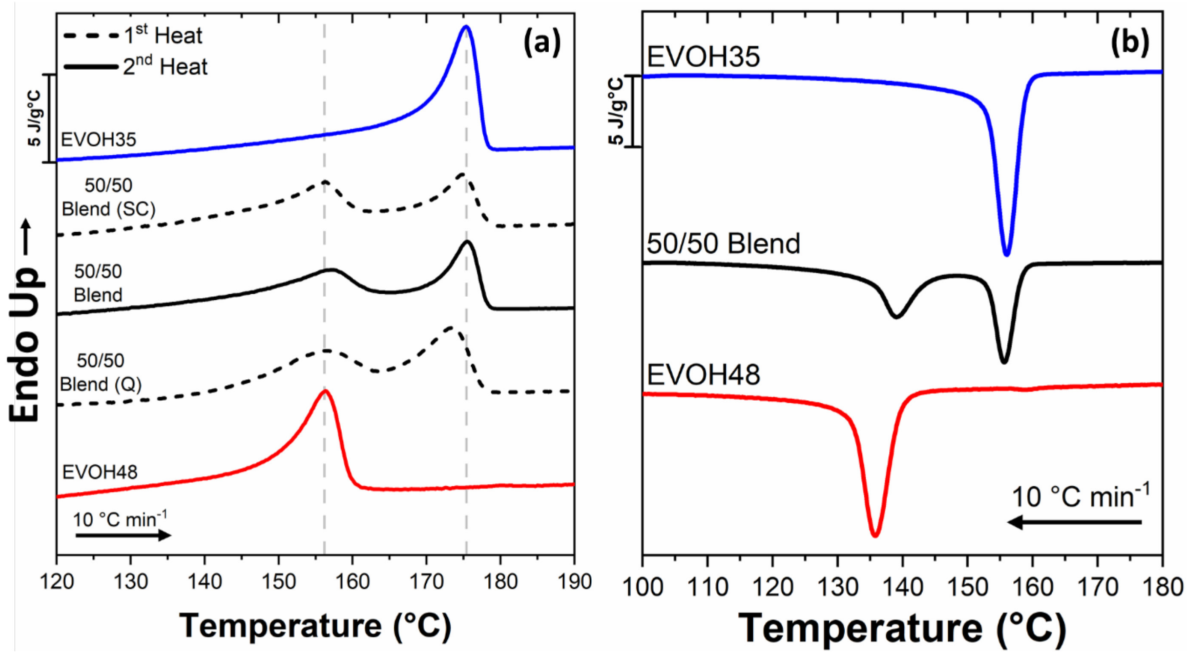

Figure S29. DSC thermograms of EVOH48-35 blend compared with individual components processed at 210 °C (EVOH48) and 200 °C (EVOH35): (a) heating curves (b) cooling curves.

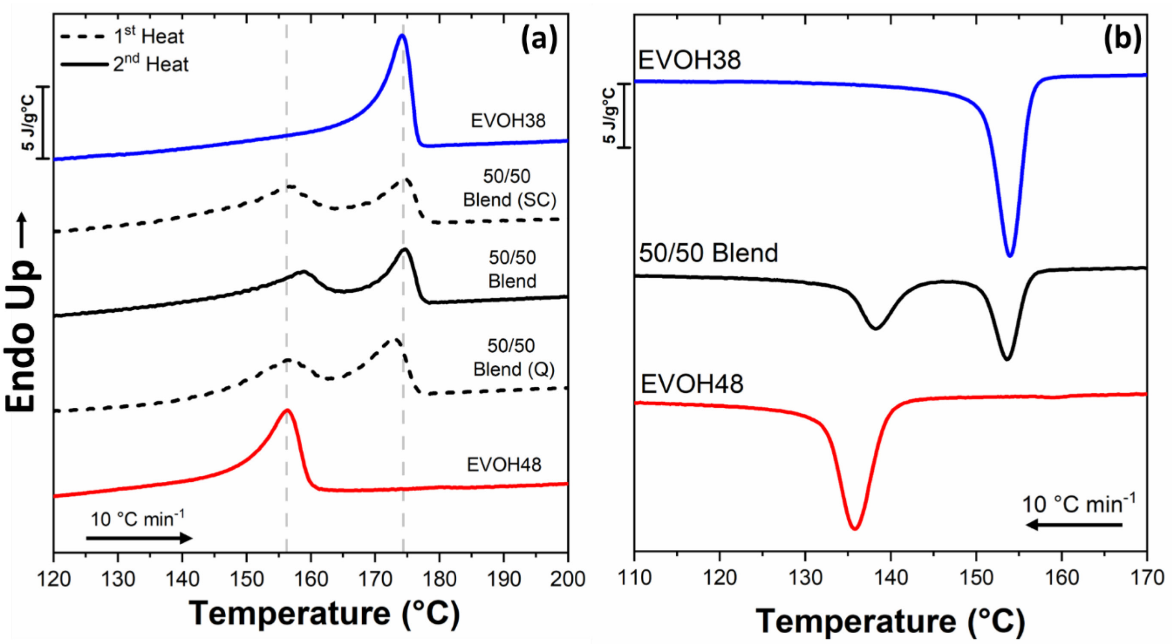

Figure S30. DSC thermograms of EVOH48-38 blend compared with individual components processed at 210 °C (EVOH48) and 190 °C (EVOH35): (a) heating curves (b) cooling curves.

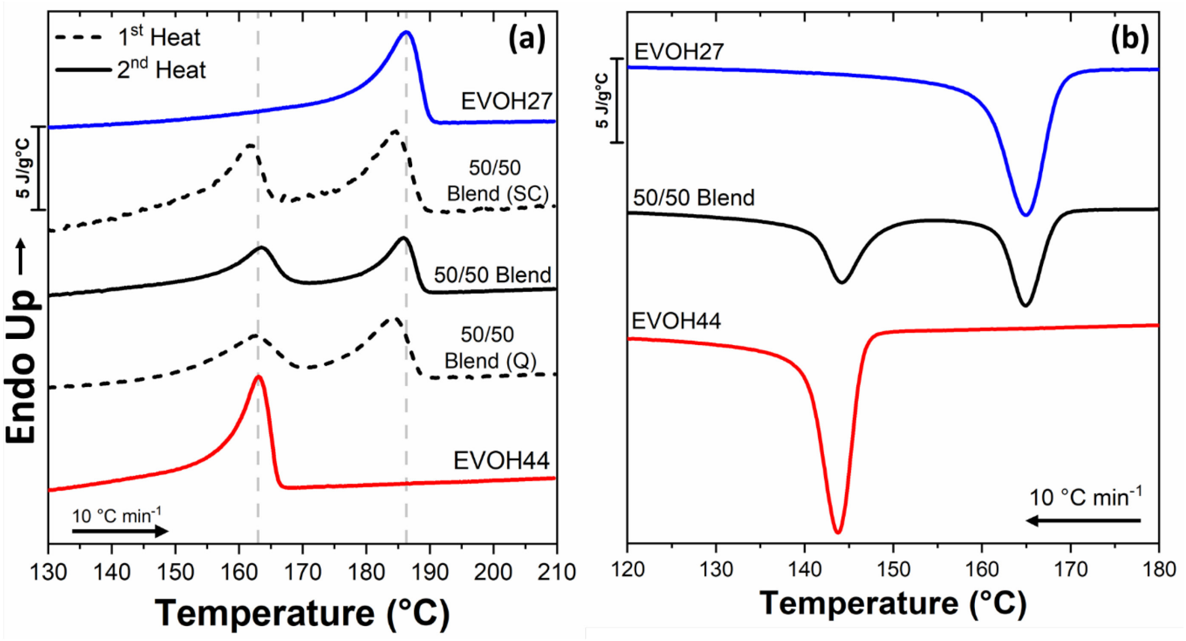

Figure S31. DSC thermograms of EVOH44-27 blend compared with individual components processed at 190 °C (EVOH44) and 210 °C (EVOH27): (a) heating curves (b) cooling curves.

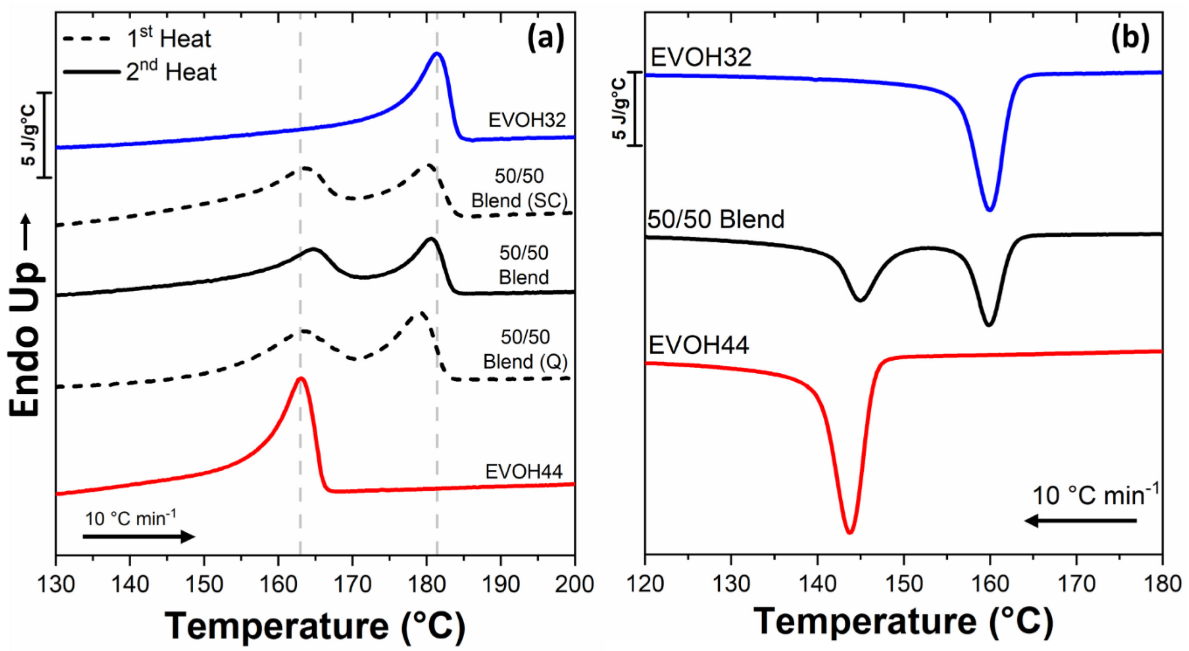

Figure S32. DSC thermograms of EVOH44-32 blend compared with individual components processed at 190 °C (EVOH44) and 200 °C (EVOH32): (a) heating curves (b) cooling curves.

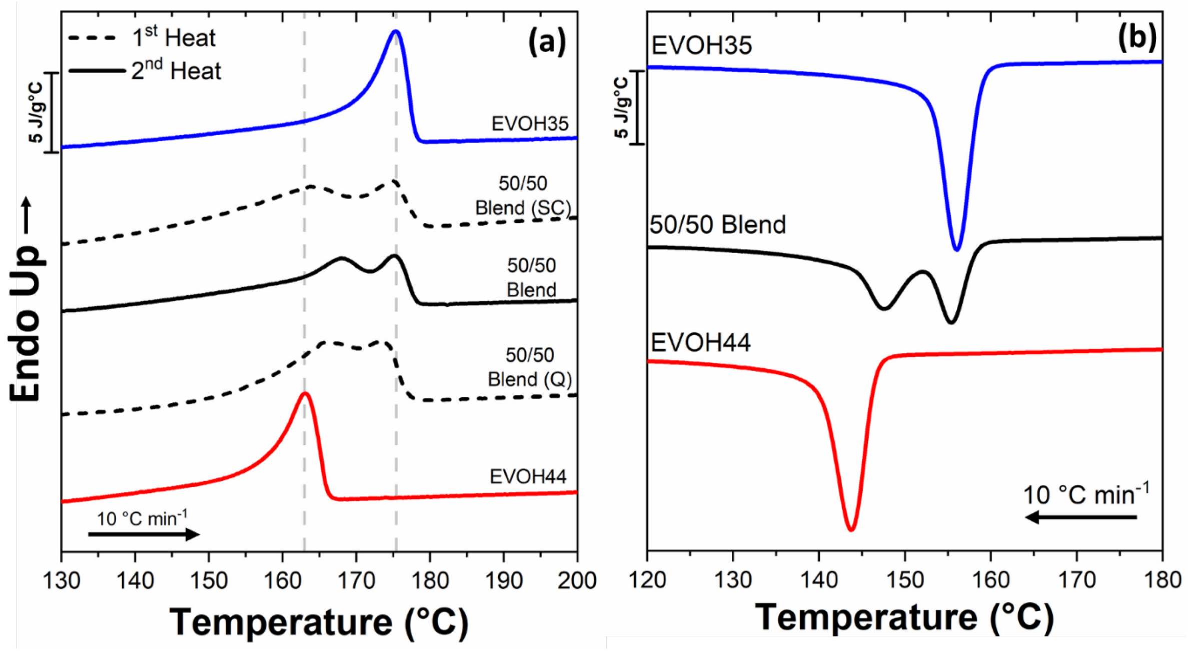

Figure S33. DSC thermograms of EVOH44-35 blend compared with individual components processed at 190 °C (EVOH44) and 200 °C (EVOH35): (a) heating curves (b) cooling curves.

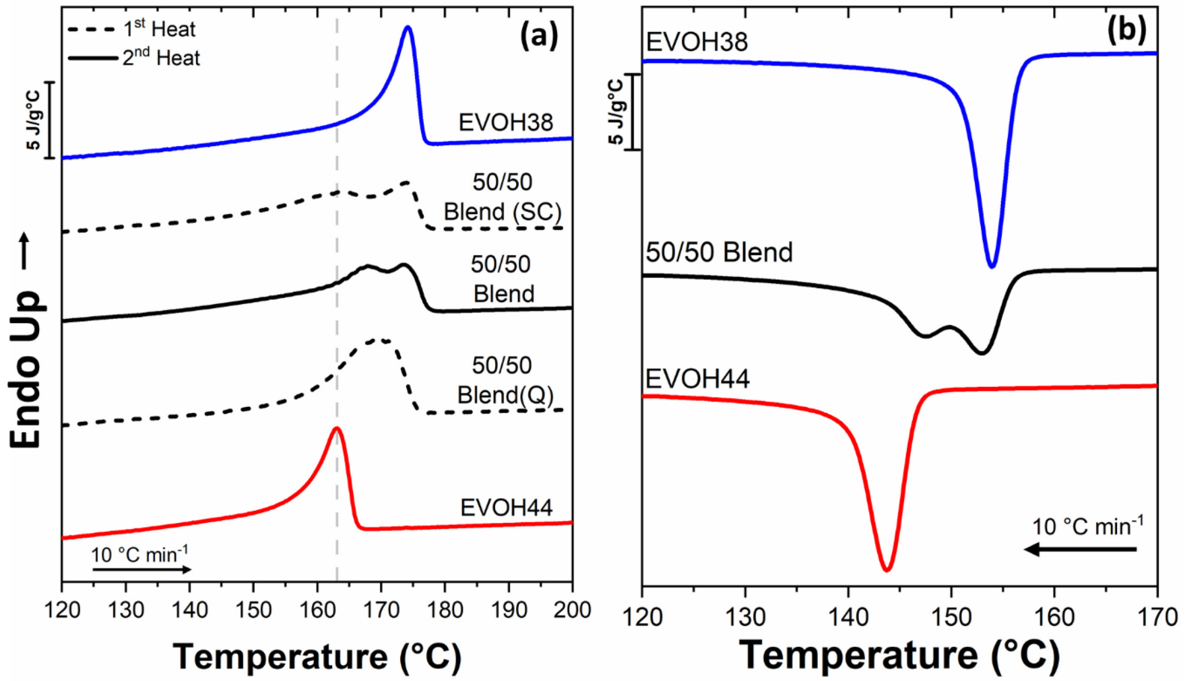

Figure S34. DSC thermograms of EVOH44-38 blend compared with individual components processed at 190 °C: (a) heating curves (b) cooling curves.

Fits to Funke et al. Experimental Values of α and β

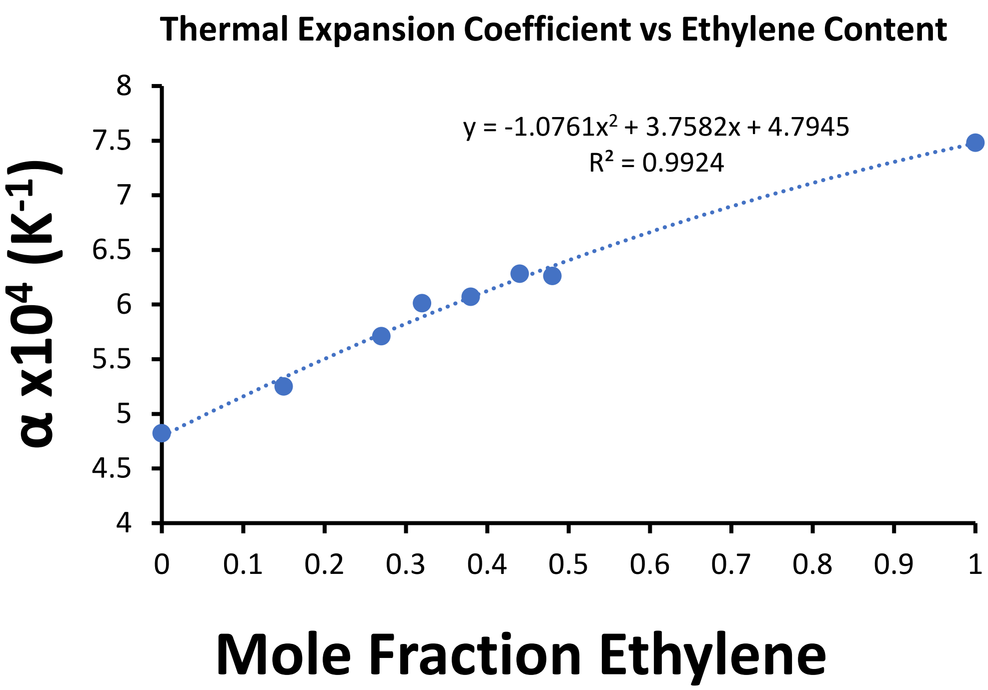

Figure S35. Quadratic curve fitted through values of thermal expansion coefficient α (at 220 °C) reported in Funke et al.[1]

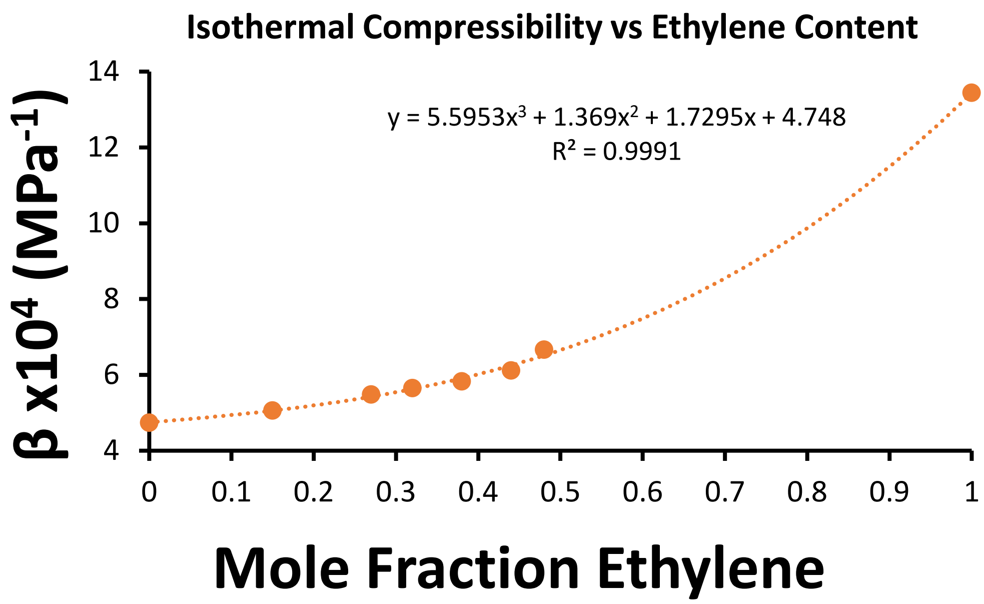

Figure S36. Cubic curve fitted through values of isothermal compressibility β (at 220 °C) reported in Funke et al.[1]

Blends of EVOH Copolymers with Polyvinyl Alcohol

Polyvinyl alcohol (Mowiol 4-98, peak T_m = 218 °C) was obtained from Sigma-Aldrich. The EVOH-PVOH blend films were prepared by solvent casting from DMSO, as described in the experimental section for EVOH blends.

The films were heated to 230 °C and held for 15 minutes and then further heated to 280 °C. From 280 °C, they were cooled at 100 °C/min to 20 °C.

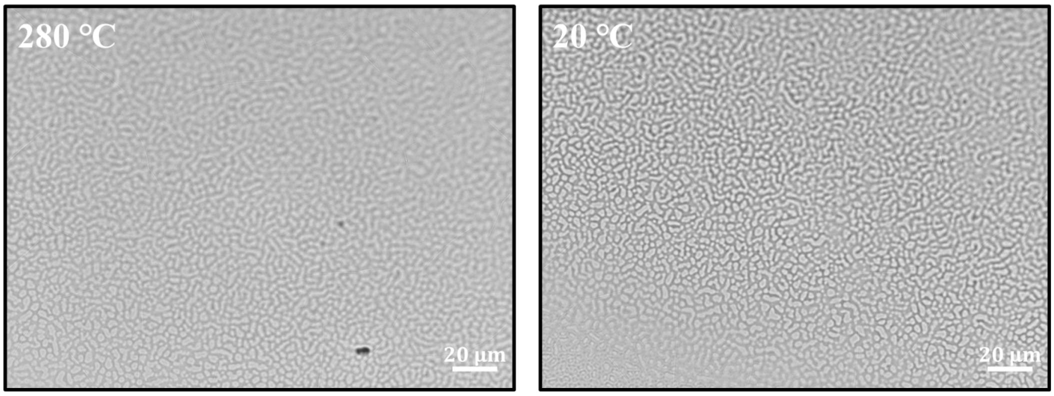

Figure S37. OM image showing EVOH27-PVOH blend as seen in the melt (a) and after cooling (b).

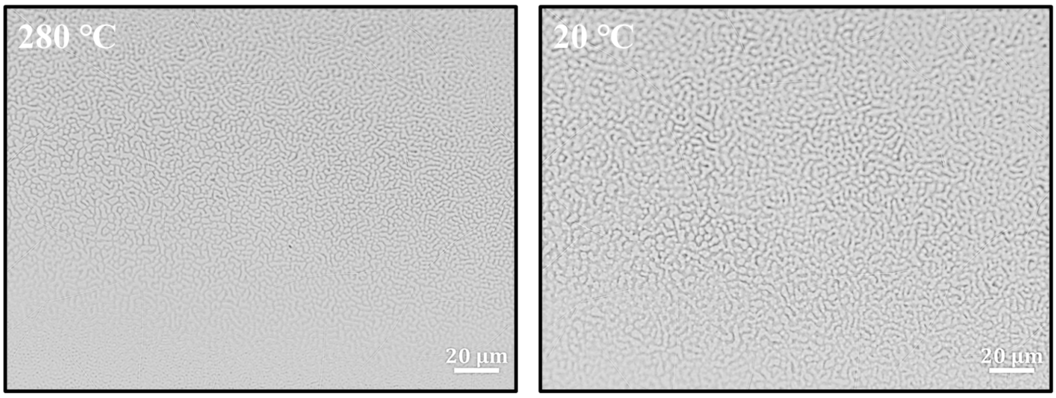

Figure S38. OM image showing EVOH32-PVOH blend as seen in the melt (a) and after cooling (b).

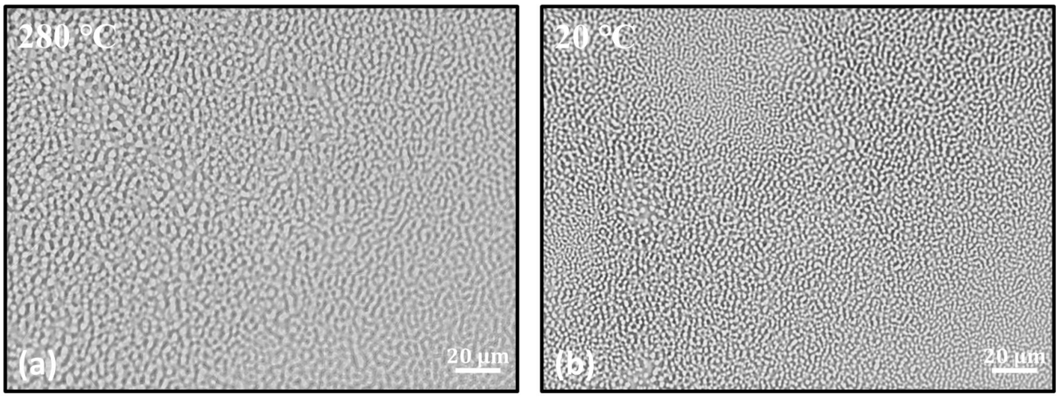

Figure S39. OM image showing EVOH35-PVOH blend as seen in the melt (a) and after cooling (b).

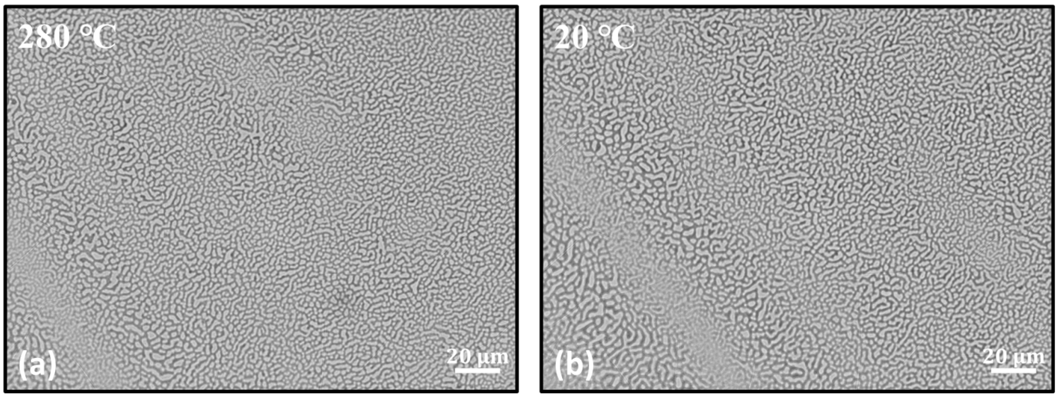

Figure S40. OM image showing EVOH38-PVOH blend as seen in the melt (a) and after cooling (b).

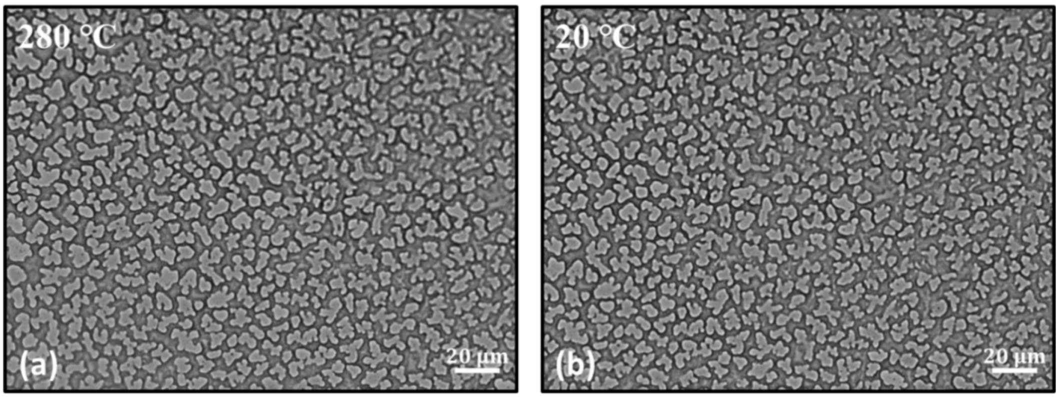

Figure S41. OM image showing EVOH44-PVOH blend as seen in the melt (a) and after cooling (b).

References

(1) Funke, Z.; Hotani, Y.; Ougizawa, T.; Kressler, J.; Kammer, H. W. Equation-of-State Properties and Surface Tension of Ethylene-Vinyl Alcohol Random Copolymers. *Eur. Polym. J.* **2007**, *43* (6), 2371–2379. https://doi.org/10.1016/j.eurpolymj.2007.03.038.
